# Senescence evolution under the catastrophic accumulation of deleterious mutations

**DOI:** 10.1093/evlett/qrad050

**Published:** 2023-11-27

**Authors:** Thomas G Aubier, Matthias Galipaud

**Affiliations:** Laboratoire Évolution and Diversification Biologique, Université Paul Sabatier Toulouse III, UMR 5174, CNRS/IRD, 31077 Toulouse, France; Department of Biology, University of North Carolina at Chapel Hill, Chapel Hill, NC, United States; Department of Evolutionary Biology and Environmental Studies, University of Zurich, Zurich, Switzerland; Department of Evolutionary Biology and Environmental Studies, University of Zurich, Zurich, Switzerland; Swiss Data Science Center, ETH, Zurich, Switzerland

**Keywords:** life history, aging, selection shadow, soma, semelparity, population regulation

## Abstract

For aging to evolve, selection against mortality must decrease with age. This prevailing view in the evolutionary theory of senescence posits that mutations with deleterious effects happening late in life—when purging selection is weak—may become fixed via genetic drift in the germline, and produce a senescent phenotype. Theory, however, has focused primarily on growing populations and the fate of single deleterious mutations. In a mathematical model, we demonstrate that relaxing both of these simplifying assumptions leads to unrealistic outcomes. In density-regulated populations, previously fixed deleterious mutations should promote the fixation of other deleterious mutations that lead to senescence at ever younger ages, until death necessarily occurs at sexual maturity. This sequential fixation of deleterious mutations is not promoted by a decrease in population size, but is due to a change in the strength of selection. In an individual-based model, we also show that such evolutionary dynamics should lead to the extinction of most populations. Our models therefore make rather unrealistic predictions, underlining the need for a reappraisal of current theories. In this respect, we have further assumed in our models that the deleterious effects of mutations can only occur at certain ages, marked, for instance, by somatic or physiological changes. Under this condition, we show that the catastrophic accumulation of deleterious mutations in the germline can stop. This new finding emphasizes the importance of investigating somatic factors, as well as other mechanisms underlying the deleterious effects of mutations, to understand senescence evolution. More generally, our model therefore establishes that patterns of senescence in nature depend not only on the decrease in selection strength with age but also on any mechanism that stops the catastrophic accumulation of mutations.

## Introduction

Aging is characterized by a gradual functional decline resulting in tissue dysfunction in multiple organs eventually causing increased mortality with age (López-Otín et al., 2013). This progressive loss of physiological integrity can be understood in light of the evolutionary theory of senescence. Namely, older individuals are usually rarer than younger ones in populations, and the strength of selection against mortality declines accordingly with age. Deleterious mutations increasing mortality late in life are therefore not efficiently purged by selection and may accumulate in the germline by genetic drift, causing a senescent phenotype (Caswell, 1978, 2001; Charlesworth, 1994; Charlesworth & Williamson, 1975; Hamilton, 1966). This classical view, complemented by the consideration that selection favors mutations that are beneficial early in life but costly late in life (a form of antagonistic pleiotropy; Williams, 1957), is still the dominant explanation for the evolution of senescence (e.g., Cheng & Kirkpatrick, 2021; Rodríguez et al., 2017; but see Baudisch, 2005; Roper et al., 2021). Albeit appealing and built on a solid mathematical foundation, this view has two main limitations: it predominantly draws conclusions assuming unconstrained population growth, and it mostly considers the fate of deleterious mutations in isolation from each other (with some notable exceptions; Charlesworth, 2001; Partridge & Barton, 1993; Wachter et al., 2013, but again, assuming unconstrained population growth). As we will highlight below, relaxing these two assumptions together leads to unrealistic predictions about the evolution of senescence.

Unconstrained population growth is a strong, simplifying assumption in a world with finite resources. Some studies have instead considered stationary populations or populations with finite sizes, thereby asserting density-dependent regulation of population growth as a major factor influencing the evolution of senescence (Abrams, 1993; André & Rousset, 2020; Dańko et al., 2018; de Vries et al., 2022; Lehtonen, 2020; Williams & Day, 2003). Strikingly, if recruitment into the adult population increases as population density decreases (e.g., because more resources are available, and fecundity or offspring survival increases accordingly), any increase in adult mortality leads to an even greater count imbalance between younger and older age classes and should favor the evolution of senescence at younger ages (Williams et al., 2006). For instance, high predation may cause populations to senesce earlier compared to those that are less subject to predation (e.g., as often hypothesized in mice vs. bats; Wilkinson & Adams, 2019).

Such developments have recently led to a new theoretical framework of senescence, on which our study is based, that focuses on lethal mutations as an illustrative limiting case (Lehtonen, 2020). Lehtonen (2020) considered a stationary population by assuming that recruitment into the adult population compensates adult mortality (e.g., via density-dependent recruitment) and derived the expression of the strength of selection acting on age-dependent lethal mutations. This model predicts the existence of a critical age, which we note $\hat{x}$, of mutation expression above which genetic drift is likely to overcome natural selection (a ‘drift barrier’; Kimura, 1962; Lynch, 2007). Any lethal mutation expressed after the critical age $\hat{x}$ can invade by genetic drift; this should eventually lead to a senescent phenotype characterized by $\hat{x}$ as a maximum life span. Indeed, given enough time, a mutation with a lethal effect occurring very close to critical age $\hat{x}$ can be expected to invade (in addition to the many mutations with lethal effects well after age $\hat{x}$), and the maximum life span thus becomes approximately $\hat{x}$ (see Supplementary Appendix A in Supplementary Material, where we present the mathematical demonstration provided by Lehtonen, 2020). Under simplifying assumptions, Lehtonen (2020) shows that this critical age is $\hat{x}=\frac{\ln\left(N_{\text{e}}\right)}{\mu }$ and therefore depends on the effective population size ($N_{\text{e}}$), such that populations with large effective population sizes are not very susceptible to genetic drift and should thus senesce at old age. Consistent with previous theory (Hamilton, 1966), $\hat{x}$ also depends on the adult mortality rate, μ, such that populations with high mortality rates (e.g., experiencing high predation) should senesce at younger ages than those with low mortality rates.

This critical age $\hat{x}$ provides a theoretical limit for maximum life spans, which can be used as a basis for empirical tests. Nonetheless, the underlying mathematical derivation is actually based on the fate of a single mutation in a nonsenescent population, thereby overlooking that senescence results from the accumulation of several deleterious mutations in germlines. This is a major simplifying assumption because, in density-regulated populations, any deleterious mutation fixed in the population increases mortality and thus changes the selection gradient on other mutations (just as higher predation does). We here address this gap using Lehtonen’s (2020) approach and show that, in density-regulated populations, the accumulation of deleterious mutations in germlines leads to senescence at younger and younger ages. This process occurs because the strength of selection on any deleterious mutation changes as mutations accumulate, and thus acts in addition to the so-called “mutational meltdown” that can occur in small populations (when the fixation of deleterious mutations decreases population size and thus increases the magnitude of genetic drift; Lynch et al., 1993, 1995). We then expand on current evolutionary theories of senescence and highlight key conditions under which such an accumulation of deleterious mutations can be halted.

## Methods

Here, we present the model and the main analytical expressions we could derive. We begin by demonstrating, with as few assumptions as possible, that lethal mutations should consistently accumulate and reduce the maximum life span. We then make the same simplifying assumptions as in Lehtonen (2020) to provide analytical expressions of the maximum life span that could evolve following the invasion of new lethal mutations, as well as long-term evolutionary outcomes depending on the genetic variation available. For the convenience of the reader, the reasoning behind these analytical derivations is explained in detail in the Results and discussion section, which can be understood without reading the Methods section.

In Supplementary Material, we detail mathematical derivations in Supplementary Appendix A, and we present and analyze an individual-based model in Supplementary Appendix B where we relax some assumptions made in our mathematical model.

### Mathematical analysis without assumptions on age-specific reproductive success: the accumulation of lethal mutations should consistently reduce maximum life span

We consider a density-regulated population, where the stationary age distribution has been reached. A stationary population implies a lifetime reproductive success, LRS, equals to one (Fisher, 1930), such that


$$\text{LRS}=\int_{x_{\text{min}}}^{X}k\,\left(t\right)\,\text{d}\,t=1,$$ (1)

where t represents age, $x_{\text{min}}\geq 0$ is the age of first reproduction, $X>x_{\text{min}}$ is the maximum age at death, and k⁢(t) is the age-specific reproductive success (with k⁢(t)>0 for $x_{\text{min}}\leq t\leq X$). We thus assume that individuals die no later than age X. This equation therefore applies to nonsenescent populations (for X=∞), as well as to already senescent populations, for example, due to a previously fixed mutation that is lethal at age X. Unlike in Lehtonen’s (2020) model, no simplifying assumption is made on the function k describing age-specific reproductive success (which results from the combination of age-specific survival and reproduction rates), except that it is positive between $x_{\text{min}}$ and X.

Following Medawar (1952) and Lehtonen (2020), we investigate a simple scenario where a senescent phenotype is caused by lethal mutations (autosomal and dominant) expressed at specific ages and spreading in the population. We consider a mutant expressing a new mutation that is lethal when age x is reached, and we assume that this age of lethality occurs between the age of first reproduction and the maximum age at death (with $x_{\text{min}}\leq x\leq X$). We extend Lehtonen, ’s (2020) model and consider that this new mutant has a different fecundity from that of wild-type individuals, as controlled by a parameter α≥0. When α=1, the mutation is not pleiotropic and leads only to death at age x. By constrast, when α<1, the new mutation also reduces fecundity, whereas when α>1, it also increases fecundity. The lifetime reproductive success, ${\text{LRS}}_{\text{mut}}$, of such a mutant is then:


$${\text{LRS}}_{\text{mut}}=\int_{x_{\text{min}}}^{x}\alpha \,k\,\left(t\right)\,\text{d}\,t.$$ (2)

The selection coefficient s⁢(x) on such mutation is expressed as the difference in lifetime reproductive success between mutant and wild-type individuals, ${\text{LRS}}_{\text{mut}}-\text{LRS}$, which leads to:


$$s\,\left(x\right)=\alpha \,\int_{x_{\text{min}}}^{x}k\,\left(t\right)\,\text{d}\,t-1.$$ (3)

The mutation is beneficial when s⁢(x)>0 and deleterious when s⁢(x)<0. Clearly, a mutation that is lethal at age x is less deleterious when lethality occurs late in life. This explains why the selection coefficient s⁢(x) increases with $x\in \left[x_{\text{min}},X\right]$, and lies in the interval [−1,s⁢(X)].

This model therefore makes different predictions depending on how the lethal mutation alters fertility. If the mutation increases fecundity (α>1), we have s⁢(X)>0 and therefore s⁢(x)>0 if x is high enough. Such a fecundity-enhancing mutation is beneficial, provided it is lethal sufficiently late in life. Now, let us consider the case where the mutation is deleterious so that s⁢(x)<0. We assume that it can invade by genetic drift when $s\,\left(x\right)>-1/N_{\text{e}}$, where $N_{\text{e}}$ is the effective population size (i.e., when the strength of purging selection is less than $1/N_{\text{e}}$; Kimura, 1962; Lehtonen, 2020; Lynch, 2007). If the mutation strongly decreases fecundity (for a very low α<1), we can have $s\,\left(X\right)<-1/N_{\text{e}}$ and therefore $s\,\left(x\right)<-1/N_{\text{e}}$ for all x. Such fertility-reducing mutation cannot invade in the population by genetic drift, whatever the age at which it is lethal. In contrast, if a mutation does not strongly decrease fecundity, so that we have $s\,\left(X\right)>-1/N_{\text{e}}$, there is a value of x, which we note ${\hat{x}}^{*}$, in the interval $[x_{\text{min}},X[$, so that $s\,\left({\hat{x}}^{*}\right)=-1/N_{\text{e}}$ (because $-1/N_{\text{e}}>-1$ if we assume that $N_{\text{e}}>1$). This means that a mutation can invade by genetic drift if it is lethal after age ${\hat{x}}^{*}$. Given that ${\hat{x}}^{*}<X$, there can always be a mutation that is lethal before age X and spreads to fixation by genetic drift, thereby lowering the maximum age at death (i.e., the maximum life span) in the population.

Some time after the fixation of a new lethal mutation by genetic drift, the lifetime reproductive success will eventually be equal to one if we assume that the population remains stationary and does not collapse (Equation 1; Fisher, 1930). This implies, for instance, that recruitment into the adult population gets higher and compensates for the death caused by the new lethal mutation. The argument above can therefore be iterated indefinitely, and the maximum age at death is predicted to progressively decrease. Of course, the iteration stops when $X=x_{\text{min}}$ and no further lethal mutation can spread. Importantly, as explained in detail later, this catastrophic accumulation of lethal mutation occurs provided that the sort of mutations under consideration arise.

### Mathematical analysis with assumptions on age-specific reproductive success

#### Maximum life span that could evolve following the invasion of a new lethal mutation

We now make the same simplifying assumptions as in Lehtonen (2020) to provide analytical expressions of the maximum life span that could evolve following the invasion of new lethal mutations. We consider that age-specific reproductive success, noted k⁢(t) above, results from the combination of age-specific survival and reproduction rates. We assume that the extrinsic mortality rate, μ, is independent of age, so that the survival probability to age t is $e^{-\mu \,t}$, and that fecundity, F, is the same at all ages. For simplicity, we also consider the case where age at first reproduction is equal to 0 (though our results do not change qualitatively when this assumption is relaxed; see also Lehtonen, 2020). Here, fecundity therefore reflects the recruitment into the adult population.

As in the demonstration above, we assume that a lethal mutation is already fixed in the population. The lethal effect of this mutation occurs at age X>0, and therefore individuals die no later than age X. Overall, we therefore assume $k\,\left(t\right)=F\,e^{-\mu \,t}$ and given that we consider a stationary population so that L⁢R⁢S=1 (Equation 1), we get $F=\frac{\mu }{1-e^{-\mu \,X}}$. The fecundity is therefore higher than in the case without any lethal mutation fixed in the population (F>μ) and compensates for the death caused by the lethal mutation already fixed in the population.

We consider mutants expressing a new mutation that is lethal when age x is reached. We extend Lehtonen’s (2020) model and consider that these mutants have a different fecundity equal to α⁢F with α≥0, as in the demonstration above. From the expression of the selection coefficient, we can show that a mutation can spread by genetic drift in the population if its lethal effect occurs after the critical age ${\hat{x}}^{*}$, so that:


$${\hat{x}}^{*}=\frac{1}{\mu }\,\ln\left(\frac{\alpha }{N_{\text{e}}\,\left(\alpha -1\right)+1+e^{-\mu \,X}\,\left(N_{\text{e}}-1\right)}\times N_{\text{e}}\right).$$ (4)

From this expression, we can show that ${\hat{x}}^{*}<X$. Therefore, if death at age X is caused by a mutation that was able to invade by genetic drift in a nonsenescent population, leading for example to $X=\hat{x}=\frac{\ln\left(N_{\text{e}}\right)}{\mu }$ (assuming that the mutation with the earliest lethal effect that can invade, as predicted by Lehtonen, 2020, eventually invades if given enough time), new lethal mutations causing death at an even earlier age can invade. This would not have been the case if the mutation causing death at age X had not invaded in the first place.

#### Long-term evolutionary outcome when lethal effects of mutations can occur at any age

After lethal mutations invading subsequently, the maximum life span will correspond to the maximum age at death below which no other lethal mutation can invade, that is, when ${\hat{x}}^{*}=X$. This occurs only when the maximum age at death approaches the age of first reproduction, which we assumed to be 0:


$$\lim_{X\to 0^{+}}{\hat{x}}^{*}=0.$$ (5)

**Figure 1:**
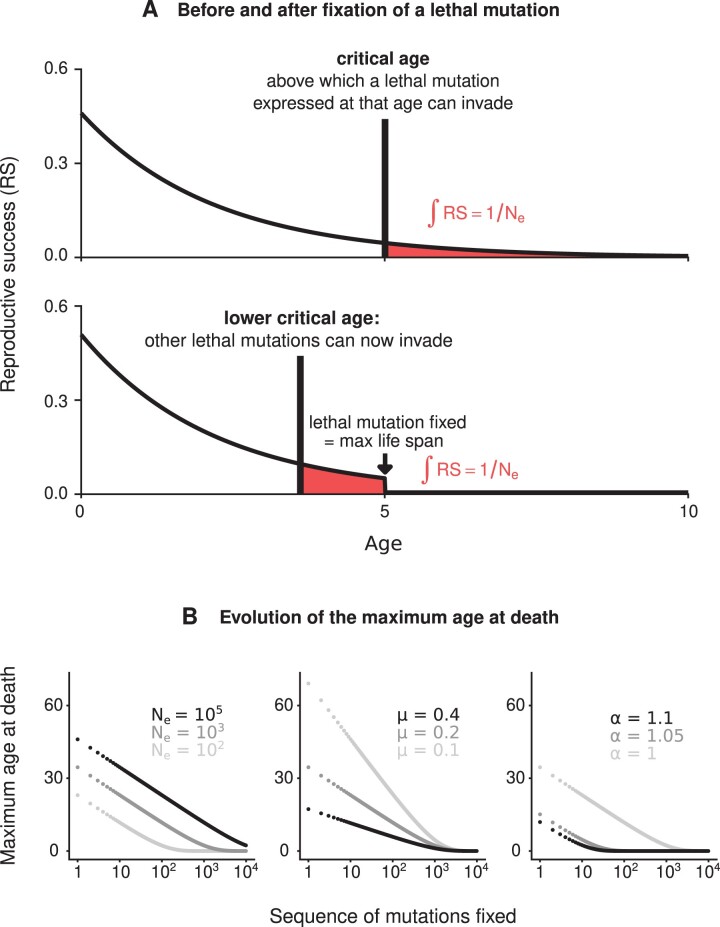
Accumulation of age-dependent lethal mutations and consequences for senescence. (A) We represent the age-specific reproductive success, defined as the chance of an individual chosen at birth reproducing at a given age. The remaining reproductive output of any organism decreases through her lifetime, and any lethal mutation expressed after a critical age, when genetic drift overcomes selection, can invade. This critical age decreases as lethal mutations get fixed, ultimately favoring the evolution of senescence at a younger age. Note the slight increase in reproductive success, especially at younger ages, when a lethal mutation gets fixed (ensuring that the growth rate remains equal to one). In (A), we use Equation 4 and parameter values are chosen for illustration purposes: $N_{\text{e}}=10$, μ=0.46 and no pleiotropy (α=1). (B) The accumulation of lethal mutations, under the simplifying assumption that each invading mutation is expressed exactly at the critical age ${\hat{x}}^{*}$, decreases the maximum age at death, until individuals die immediately after reaching sexual maturity (at age zero here). All else being equal, the maximum age at death decreases as the effective population size decreases (low $N_{\text{e}}$), as the adult mortality rate increases (high μ), and as the pleiotropic effect increases (high α). In (B), we use Equation 4 and baseline parameter values are: $N_{\text{e}}=10^{3}$, μ=0.2 and α=1.

Lethal mutations will keep invading and will have lethal effects at earlier ages until the maximum age at death approaches the age of first reproduction. This is confirmed using numerical iterations where we evaluate the accumulation of lethal mutations, assuming that mutations that subsequently invade have a lethal effect at the critical age ${\hat{x}}^{*}$ assessed at the current maximum age at death (Figure 1B)

#### Long-term evolutionary outcome when lethal effects of mutations can only occur at certain ages

**Figure 2:**
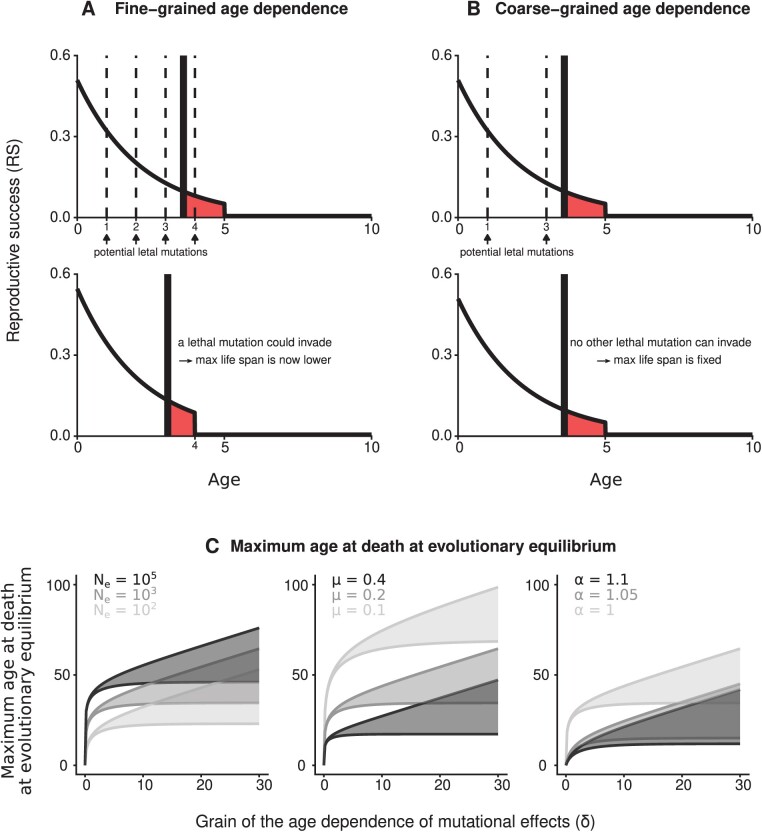
The effect of the grain of the age dependence of mutational effects on the accumulation of lethal mutations, and consequences for senescence. (A) If at least one of the deleterious mutational effect occurs after the critical age, then a lethal mutation can ultimately invade, decreasing the maximum age at death. (B) If not, then the maximum age at death is fixed. Therefore, the grain of the age dependence of deleterious mutational effects determines at which age the accumulation of lethal mutation stops. In (A) and (B), we use Equation 4, and parameter values are chosen for illustration purposes: $N_{\text{e}}=10$, μ=0.46 and no pleiotropy (α=1). (C) A coarse-grained distribution of mutational effects ultimately leads to a higher maximum age at death. The maximum age at death at evolutionary equilibrium decreases as the effective population size decreases (low $N_{\text{e}}$), as the adult mortality rate increases (high μ), and as the pleiotropic effect of the lethal mutations increases (high α). It also strongly depends on parameter δ, which is the grain of the age dependence of deleterious mutational effects (the lethal effects of mutations can occur at any of the ages separated by an age period δ; for example, δ=1 and δ=2 in (A) and (B), respectively). In (C), we use Equation 6 and baseline parameter values are: $N_{\text{e}}=10^{3}$, μ=0.2, and α=1.

We now assume that the lethal effects of mutations can occur at any age separated by an age period δ (e.g., as in Figure 2A and B). In other words, we keep assuming that the effect of each mutation occurs at a given age, but we now consider this to be the case only at certain ages separated by an age period δ. We thus consider how different grains, defined as regular age intervals at which mutational effects can occur, affect the evolution of senescence. Parameter δ therefore describes the grain of the age dependence of deleterious effects.

In this case, the last mutation able to invade is the one that has a lethal effect within the age interval $\left[X^{†}-\delta ,X^{†}\right]$, so that $X^{†}$ is X solution to $X-{\hat{x}}^{*}=\delta$. We get the expected maximum age at death at evolutionary equilibrium within the interval, as shown in Figure 2C:


$$\left[\frac{1}{\mu }\,\ln\left(\frac{\alpha \,N_{\text{e}}-e^{-\mu \,\delta }\,\left(N_{\text{e}}-1\right)}{N_{\text{e}}\,\left(\alpha -1\right)+1}\right),\frac{1}{\mu }\,\ln\left(\frac{N_{\text{e}}\,\left(\alpha \,e^{\mu \,\delta }-1\right)+1}{N_{\text{e}}\,\left(\alpha -1\right)+1}\right)\right].$$ (6)

The value of the maximum age at death at evolutionary equilibrium reached within this interval depends on the age at which the deleterious effect of the last lethal mutation that invaded the population occurs.

## Results and discussion

### The fixation of a senescence-inducing germline mutation favors the evolution of even earlier senescence

As detailed in the Methods, following Medawar (1952) and Lehtonen (2020), we consider a simple scenario where a senescent phenotype is caused by the expression of lethal mutations expressed at specific ages and spreading in the population by genetic drift. Instead of focusing on the fate of a single lethal mutation, we consider the accumulation of lethal mutations as a sequential process, in which each mutation has a chance to become fixed before another mutation occurs. As shown by Lehtonen (2020), a senescent phenotype can initially result from the invasion of a mutation that is lethal late in life into a nonsenescent population. Strikingly, we show that the fixation of any lethal mutation can promote the invasion of other lethal mutations that cause senescence earlier in life (Equations 3 and 4; as illustrated in Figure 1A). This occurs because the fixation of a lethal mutation increases adult mortality, which is then compensated by an increase in recruitment into the adult population (either through higher adult fecundity or higher offspring survival to sexual maturity) so that the population remains stationary. The population regulation that follows the fixation of a lethal mutation therefore leads to a greater count imbalance between younger and older age classes. In turn, this change in the selection gradient with age favors the fixation of other lethal mutations that cause senescence even earlier in life. Importantly, we assume that the effective population size, $N_{\text{e}}$, is fixed. Therefore, the feedback loop we highlight involves a reduction in the strength of selection purging deleterious mutations acting late in life and not a reduction in the magnitude of genetic drift, as posited by the mutational meltdown theory (Lynch et al., 1993, 1995).

In contrast to a purely deleterious mutation, a lethal mutation that increases fecundity (a pleiotropic mutation) can invade either by genetic drift or by selection; the latter occurs when mutants enjoy an increase in fecundity high enough to compensate for an early death (see Methods). As with a purely deleterious mutation, we show that the invasion of a pleiotropic mutation is more likely if another lethal mutation has already been fixed in the population. We also show that the same rationale applies to nonlethal mutations, whether purely deleterious or pleiotropic (Supplementary Appendix A in Supplementary Material).

### The sequential accumulation of germline mutations consistently leads to semelparity

Over evolutionary time, the maximum age at death should decrease, regardless of the current maximum age at death in the population. Therefore, the sequential accumulation of lethal mutations should lead to a sequentially lower maximum age at death, until individuals die immediately after reaching sexual maturity (at age 0 in Figure 1B). In simulations performed using our mathematical model (Equation 4), the speed at which the maximum age at death decreases depends critically on demography (Figure 1B). All else being equal, the maximum age at death is small if the effective population size is low, which is similar to previous results from Lehtonen’s (2020) model. In addition, a higher adult mortality rate and a greater pleiotropic effect of lethal mutations also lead to a smaller maximum age at death. In all cases, however, the sequential accumulation of lethal mutations inevitably leads to a lower and lower maximum age at death (Figure 1B).

Our mathematical model thus predicts that senescence evolution should lead to semelparity, that is, a life history characterized by death shortly after first reproduction, as opposed to iteroparity. Therefore, in our model, semelparity emerges following the evolution of senescence, and without any benefit as invoked in Cole’s classic paradox, according to which only a tiny fertility advantage should favor the evolution of semelparity over iteroparity if fertility and mortality are independent of age (Charnov & Schaffer, 1973; Cole, 1954). Previous theoretical studies have already invoked the evolutionary theory of senescence in growing populations to explain semelparity, but on the assumption that reproduction already stops after a certain age, or that early fecundity evolves at the expense of late fecundity (Penna et al., 1995; Tuljapurkar, 1997). Here, we show that in density-regulated populations, semelparity should readily evolve as a result of senescence evolution, even though fecundity initially occurs at all ages and is not subject to evolution. The underlying processes are the same as in previous studies (purging selection and a change in the selection gradient; Penna et al., 1995; Tuljapurkar, 1997). In our model, however, the evolution of semelparity is driven by a demographic process widespread in nature, namely population regulation. Importantly, note that the evolutionary outcome we predict is different from the evolution of a “wall of death” (also called “catastrophic senescence”), discussed as a contrast to the observation of late-life mortality plateau (Charlesworth & Partridge, 1997; Mueller & Rose, 1996; Partridge & Barton, 1993), because we here highlight that in density-regulated populations, the fixation of deleterious mutations can promote the invasion of deleterious mutations expressed even earlier in life, not just those expressed later in life.

The evolutionary process we highlight is a positive feedback relying on changes in reproductive values, and could therefore play a role in the evolution of other traits. In our model, a lethal mutation can become fixed by genetic drift when lethality occurs at an advanced age for which the reproductive value is low enough. Because population regulation compensates for increased mortality, the fixation of this mutation eventually reduces the reproductive values at old ages relative to that at early ages. Which favor the evolution of senescence at younger ages. Therefore, any trait evolving due to a low reproductive value and reducing further reproductive value can lead to a similar feedback as in our model. This could conceivably occur in the case of reproductive senescence described as the decline in reproductive success with increasing age (but see Lemaître & Gaillard, 2017), or in the case of other traits characterizing life history, such as age-, size-, or stage-specific patterns of development, growth, and maturation. Likewise, this could occur in the case of traits not involved in life history, such as age-dependent altruism (as discussed in Rodrigues & Gardner, 2022). Whether the feedback occurs as strongly as in our model will depend on how age-specific reproductive values change as these traits evolve; this calls for further theoretical investigations.

Our mathematical model relies on simplifying assumptions that we relax in an individual-based model, presented and analyzed in Supplementary Appendix B in Supplementary Material. In particular, in this individual-based model, the fixation of deleterious mutations results from changes in frequency over many generations, unlike in our mathematical model where we have not considered the stochastic nature of drift. Simulations reveal a similar dynamic of mutation accumulation when mutations are lethal and also show that nonlethal deleterious mutations can eventually accumulate in germlines, and their combined effects also lead to an ever-lower maximum age at death.

In our mathematical model, we also simply assume that new mutations cannot cancel the lethal effects of already fixed mutations, but can only lead to an early death. In other words, we assume that there is no beneficial mutation that can lead to a later maximum age at death. This assumption makes biological sense when considering that the vast majority of mutations are either deleterious or neutral to survival, many of which may accumulate in germlines over time when their effects occur after the maximum age at death (leading to a “wall of death”). In addition, a single sporadic mutation reversing the deleterious effect of a mutation is unlikely to have a significant impact on survival in the face of the combined lethal effect of all other fixed deleterious mutations (Laird & Sherratt, 2010; Stearns, 1976; Williams, 1957). Indeed, when we relax this assumption in our individual-based model, we show that (beneficial) mutations that remove the deleterious effect of previously fixed mutations have little impact on the dynamics of mutation accumulation. Note, however, that such reverse mutations differ from beneficial mutations that increase life span by a fixed amount, independent of the deleterious mutation fixed in populations; in this latter case, which seems less plausible, the fixation of independent beneficial mutations prevents the catastrophic accumulation of deleterious mutations (as shown in simulations in Lehtonen, 2020).

### The sequential accumulation of germline mutations leads to population collapse when population regulation reaches its limit

Population regulation is a major factor explaining our results. Through density-dependent recruitment into the adult population, increased mortality caused by the expression of a previously fixed deleterious mutation changes the selection gradient on other mutations and necessarily favors the evolution of earlier senescence. In contrast, when population growth is not regulated, the accumulation of deleterious mutations rarely leads to ever earlier senescence (called “equilibrium collapse” when it occurs; Charlesworth, 2001; Wachter et al., 2013). Even in ever-growing populations, late-life mortality can alter the selection gradient, such that earlier senescence is favored (Caswell & Shyu, 2017), but this effect is much less pronounced than in density-regulated populations.

Given the critical effect of density regulation on our model outcomes, one could expect that any mechanism limiting the recruitment of young individuals into adult classes should halt the sequential evolution of a lower maximum age at death. This is all the more interesting to study that population regulation should often reach its limit in nature. The range of naturally achievable fertility rates is limited, such that a given organism can only produce a limited number of offspring in her lifetime. In this case, there is a mortality rate above which there is no increase in recruitment that can compensate for the number of deaths to maintain a stable population size. With our mathematical model, we show that when recruitment is limited, the accumulation of deleterious mutations should indeed cease to lead to senescence at ever younger ages (Supplementary Appendix A in Supplementary Material). Nonetheless, in our individual-based model (Supplementary Appendix B in Supplementary Material), we show that with such limited recruitment, populations inevitably collapse after reaching a certain maximum age at death, due to incomplete compensation for mortality. This is not so much of a surprise, because assuming limited recruitment implies that the population growth rate is less than one (as shown in our mathematical model, in Supplementary Appendix A in Supplementary Material).

Living organisms are obviously not all doomed to die shortly after they reach sexual maturity, that is, organisms are not all semelparous, as predicted by our mathematical model. Similarly, lineages of iteroparous organisms (reproducing repeatedly during their lives) are not all doomed to go extinct due to the evolution of senescence, as predicted by our individual-based model. It is therefore doubtful that the processes described above consistently happen in nature. We now address this inconsistency by showing that such an accumulation of deleterious mutations can be stopped if deleterious effects of mutations occur at certain ages only, marked for instance by important physiological changes (including those associated with senescence itself).

### The age dependence of mutational effects may determine the cessation of mutation accumulation

Classical models of senescence assume that mutations have age-specific effects, but they rarely address the mechanisms involved in this time dependence. Surely, the deleterious effects of mutations cannot be strictly *time* dependent (Kirkwood, 1977; Kirkwood & Melov, 2011). There should instead be correlates of time, constituting cues for gene expression or enhancers of mutational effects. Recent studies, which form the basis of the “early-life inertia” theory of aging, provide an ideal framework for understanding the timing of deleterious effects of mutations (Flatt & Partridge, 2018; Maklakov & Chapman, 2019).

The “early-life inertia” theory states that genes that are beneficial early in life may become superfluous and even deleterious when expressed later in life, due to changes in the soma. For example, genes involved in major signaling pathways regulating development have been shown to become deleterious when expressed after development has ceased, so that their down-regulation in adults results in a significant increase in life span, with no obvious cost to reproduction (Carlsson et al., 2021; Lind et al., 2021; Maklakov & Chapman, 2019). Likewise, dietary restrictions increase life span in some species, indicating potentially deleterious effects of sustained activation of nutrient signaling pathways throughout life (Mair & Dillin, 2008). Other somatic changes resulting from loss of function or the accumulation of metabolic products (e.g., changes in the level of aberrant proteins or hormones; Junnila et al., 2013; Komatsu et al., 2006) may also correlate with time (albeit sometimes imperfectly) and may trigger the detrimental effects of mutations (Chia, 2014). Therefore, when deleterious effects of germline mutations occur only around a given age, it is likely not because age itself affects gene expression, but rather because the mutant’s internal (or external) environment has changed over the course of her life (i.e., over time), triggering the deleterious effect of the mutation (Kirkwood, 1977).

Recognizing that the deleterious effects of mutations depend on somatic state has consequences for how we view and model the evolution of senescence. Importantly, there must be some ages at which deleterious mutational effects are more likely than others. In contrast, the mathematical model we have presented so far—like most previous models of senescence—assumes a continuous time and considers that deleterious effects of mutations can occur at any age. This simplification, which has already been identified before (Lehtonen, 2020), means that in an already senescent population, a new mutation with an earlier lethal effect may always appear, whether it is 1 hr, 1 s, or in fact any infinitesimal time before the current maximum age at death. As long as the new lethal effect occurs after the critical age above which genetic drift overcomes selection, the mutation can eventually spread in the population, leading to an earlier (albeit slightly) maximum age at death (Figure 1A). Now assume that no harmful effects of mutations can occur until 10 years of age, when somatic changes occur in the organism and trigger deleterious mutational effects. In this context, the maximum age at death simply cannot evolve to be less than 10 years.

These are extreme but revealing examples. With our mathematical model, we next investigate the accumulation of mutations in the germline when the timing of mutational effects is not continuous throughout life. We keep assuming that the effect of each mutation occurs at a given age, but we now assume that mutations can only be deleterious at certain ages. We consider how different grains, δ, defined as regular age intervals at which mutational effects can occur, affect the evolution of senescence. As long as mutational effects can occur after the critical age, which is likely under fine-grained distributions of mutational effects, new deleterious mutations can fixate in the germline, resulting in an earlier maximum age at death (Figure 2A). By contrast, when mutational effects cannot occur after the critical age because of a coarse-grained distribution of mutational effects, then no new mutation can fixate and the maximum age at death can no longer evolve towards younger ages (Figure 2B). A coarse-grained distribution of mutational effects effectively prevents deleterious mutations from accumulating at ever younger ages (Figure 2B). Under this new formalism, the maximum age at death at evolutionary equilibrium is still lower as the effective population size decreases, as the adult mortality rate increases, and as the pleiotropic effect of lethal mutations increases (Equation 6; Figure 2C). This is mediated by a change in the balance between genetic drift and purging selection, as in classical evolutionary theories of senescence. Nonetheless, we highlight here that differences in senescence patterns among organisms are also likely due to differences in the age dependence of mutational deleterious effects, with coarser-grained distributions of mutational effects (higher δ) being associated with longer-lived species.

The analysis based on the mathematical derivations presented above shows that the age dependence of mutational effects can put a halt to mutation accumulation. Likewise, in Supplementary Appendix B in Supplementary Material, we could show using our individual-based model that deleterious mutations that can be expressed at the onset of each day can accumulate until the population goes extinct (because population regulation reaches its limit). By contrast, deleterious mutations that can be expressed only at the onset of each year stop accumulating at some point and determine the maximum age at death (i.e., the senescence phenotype).

These analyses remain based on very simplistic assumptions regarding the age dependence of mutational effects. That is why we also consider in our individual-based model that the expression of mutations depends on the somatic state changing stochastically over time (see Supplementary Appendix B in Supplementary Material). We show that the accumulation of deleterious mutations occurs and that the resulting maximum age at death depends on the rate at which somatic states change.

## Conclusion

**Figure 3:**
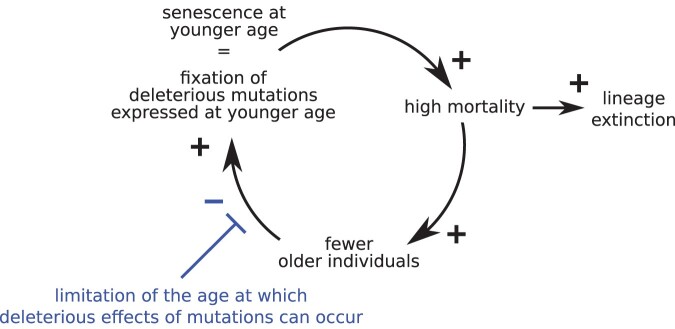
Summary scheme. The accumulation of deleterious mutations causes the evolution of senescence at a younger and younger age, until the population becomes semelparous or extinct. The limitation of the ages at which deleterious effects of mutations occur can stop the accumulation of these mutations.

In his seminal paper, Hamilton (1966) recognized that senescence “will be moulded by natural selection depend[ing] on what sort of genetical variation is available.” Indeed, senescence depends on the nature of deleterious mutations (Baudisch, 2005) and the proportion of deleterious mutations to beneficial mutations (Lehtonen, 2020). Our study also highlights the importance of this statement, but this time by showing that when all possible forms of age-dependent mutational effects exist (i.e., when genetic variation is not constrained), deleterious mutations accumulate in germlines leading to ever-lower maximum age at death (schematized in Figure 3). We thereby expose a serious inconsistency of evolutionary theories of senescence: all density-regulated populations should be doomed either to be semelparous or to collapse due to the accumulation of deleterious mutations. This inconsistency underlines the importance of considering the conditions under which the deleterious effects of mutations occur (Figure 3). We thus believe that the simple view of mutations with deleterious effects occurring at any given age (possibly in addition to other beneficial effects) should give way to theories considering the mechanisms behind the age dependence of mutational effects.

The age dependence of mutational effects can be contingent on somatic states, so that mutated genes are expressed only under certain somatic conditions, such as disease (Rodriguez-Esteban & Jiang, 2017), changes in energy conditions (Wagner, 2005), or when the soma undergoes functional changes with age (Henry et al., 2010). Alternatively, mutations can be expressed consistently throughout life, but their effect may only be felt under certain conditions (somatic or external) (e.g., as shown in *Caenorhabditis elegans*; Carlsson et al., 2021; Curran & Ruvkun, 2007; Dillin et al., 2002; Lind et al., 2021). The latter is a central tenet of the “early-life inertia” theory of senescence, which links the development of an organism to its senescence and has gained recent popularity (Flatt & Partridge, 2018; Maklakov & Chapman, 2019). Because somatic triggers of genetic effects limit the possible ages at which deleterious mutational effects occur, our results give theoretical credit to the “early-life inertia” theory, which must therefore be considered in parallel with evolutionary theories of senescence.

We are in need of studies explicitly considering biologically relevant stage structure (rather than age structure) with conceivable effects on the impact of deleterious mutations to understand the origin of the diversity of senescence patterns in nature (Roper et al., 2021). Our study also strongly encourages further phenotypic studies describing the conditions triggering the deleterious effects of genes degrading organ integrity and regeneration (Eguchi et al., 2019), as this is a key component of the aging process. In fact, from the above demonstration, we can conclude that evolutionary theories of senescence which ignore (somatic) triggers of genetic effects at best offer only a partial understanding of senescence evolution, and at worst, predict unrealistic outcomes.

## Supplementary Material

qrad050_suppl_Supplementary_Material

## Data Availability

All analytical derivations of the mathematical model are detailed in Appendices in Supplementary Material. The C++ and R scripts used for the individual-based simulations (not detailed in the main text), and the figures are available in a Zenodo repository (doi:10.5281/zenodo.8392458; https://zenodo.org/record/8392458).
