# Supplementary material for: Senescence evolution under the catastrophic accumulation of deleterious mutations
Source: Evol Lett. 2023 Nov 27;8(2):212–21. doi: 10.1093/evlett/qrad050 (PMC10959475; doi:10.1093/evlett/qrad050)
Supplement: qrad050_suppl_Supplementary_Material [file qrad050_suppl_supplementary_material.pdf]

Supplementary Material for:

Senescence evolution under the catastrophic accumulation of deleterious mutations

Thomas G. Aubier, Matthias Galipaud

in *Evolution Letters*

Contents

Pages 2 - 16: Appendix A: Mathematical model and analytical derivations

- Page 2 A1. Conditions of invasion of a lethal mutation (Lehtonen 2020)
- Pages 3 - 5 A2. Conditions of invasion of a lethal mutation affecting fecundity
- Pages 6 - 10 A3. Conditions of invasion with a lethal mutation fixed in the population
- Pages 11 - 13 A4. Conditions of invasion with a lethal mutation fixed in the population when recruitment is limiting
- Pages 14 - 16 A5. Selection purging many deleterious mutations is getting weaker as any other deleterious mytations gets fixed

Pages 17 - 30: Appendix B: Individual-based model

- Pages 17 - 19 B1. The model
- Pages 19 - 20 B2. Simulation experiments
- Page 20 B3. Results
- Pages 21 - 31 B4. Figures

Appendix A: Mathematical model and analytical derivations

A1. Conditions of invasion of a lethal mutation (Lehtonen, 2020)

We consider the case where age at first reproduction is equal to 0, and where the stationary age distribution has been reached. Extrinsic mortality, μ, is independent of age, so that survival to age t is $l(t) = e^{-\mu t}$. Fecundity, F, is the same at all ages.
A stationary population implies a lifetime reproductive success equals to one:

$$\mathrm{LRS} = \int_0^{+\infty} F.\,l(t)\,\mathrm{d}t = 1 \tag{1}$$

Thus:

$$F = \frac{1}{\int_0^{+\infty} l(t)\,\mathrm{d}t} \tag{2}$$

In the case without any deleterious mutation fixed in the population, we have:

$$F = \frac{1}{1/\mu} = \mu \tag{3}$$

Then, the lifetime reproductive success of a mutant expressing a lethal mutation at age x is:

$$\mathrm{LRS_{mut}} = \int_0^{x} F.\,l(t)\,\mathrm{d}t \tag{4}$$

And therefore:

$$\mathrm{LRS_{mut}} = \int_0^{x} \mu.\,e^{-\mu t}\,\mathrm{d}t = \left[-e^{-\mu t}\right]_0^x = 1 - e^{-\mu x} \tag{5}$$

The selection coefficient $s(x)$ against such lethal mutation expressed at age x is expressed as:

$$s(x) = \mathrm{LRS_{mut}} - \mathrm{LRS} \tag{6}$$

And here:

$$s(x) = -e^{-\mu x} \tag{7}$$

A lethal mutation can spread over the population when $|s(x)| < 1/N_\mathrm{e}$, with N_e the effective population size. The age $\hat{x}$ from which a deleterious mutation can spread (drift barrier) is found by solving:

$$|s(\hat{x})| = e^{-\mu\hat{x}} = \frac{1}{N_\mathrm{e}} \tag{8}$$

Therefore:

$$\boxed{\hat{x} = \frac{\ln(N_\mathrm{e})}{\mu}} \tag{9}$$

Any lethal mutation expressing after age $\hat{x}$ can spread in the population via genetic drift. This critical age $\hat{x}$ can be considered as representing the maximum life span that can evolve. Indeed, assuming enough time, mutations with lethal effects from age $\hat{x}$ to infinity are predicted to invade by genetic drift, such that the earliest-acting of the fixed lethal mutations is expected to have an effect at an age greater than $\hat{x}$ but arbitrarily close to $\hat{x}$. The maximum life span has therefore become approximately $\hat{x}$. Importantly, this relies on the assumption that the selection gradient does not change as mutations accumulate,

A2. Conditions of invasion of a lethal mutation affecting fecundity

Again, fecundity, F, is the same at all ages, and a stationary population implies a lifetime reproductive success equals to one, leading to:

$$F = \mu \tag{10}$$

This time we consider mutants expressing a lethal mutation at age x, and that also have a different fecundity $= \alpha F$ with $\alpha \in [0, +\infty[$. When $\alpha < 1$, the mutation reduces fecundity, whereas when $\alpha > 1$, the mutation increases fecundity. For $\alpha \neq 1$, the mutation is therefore pleiotropic. The lifetime reproductive success of such mutant is:

$$\mathrm{LRS}_{\mathrm{mut}} = \int_0^x \alpha\, F.\, l(t)\, \mathrm{d}t \tag{11}$$

In the case without any deleterious mutation fixed in the population, we have $F = \mu$, and therefore:

$$\mathrm{LRS}_{\mathrm{mut}} = \alpha \int_0^x \mu.\, e^{-\mu t}\, \mathrm{d}t = \alpha \left(1 - e^{-\mu x}\right) \tag{12}$$

The selection coefficient $s(x)$ associated with such lethal mutation expressed at age x, and affecting fecundity by a factor α is expressed as:

$$s(x, \alpha) = \mathrm{LRS}_{\mathrm{mut}} - \mathrm{LRS} \tag{13}$$

And here:

$$s(x, \alpha) = \alpha \left(1 - e^{-\mu x}\right) - 1 \tag{14}$$

In contrast to a purely deleterious mutation, selection can either favor or inhibit the invasion of this mutation, i.e., $s(x, \alpha)$ can be positive or negative.

Selection favors the invasion of the mutation

Selection favors the invasion of this mutation when $s(x, \alpha) > 0$, which occurs when:

$$\alpha \left(1 - e^{-\mu x}\right) - 1 > 0 \tag{15}$$

$$x > \frac{1}{\mu} \ln \left(\frac{\alpha}{\alpha - 1} \right) \tag{16}$$

Therefore, there are x values such as $s(x, \alpha) > 0$ only for $\alpha > 1$ (otherwise, $\frac{\alpha}{\alpha-1} \leq 0$, and $\ln\left(\frac{\alpha}{\alpha-1}\right)$ is not defined). Thus, selection can favor the invasion of a pleiotropic mutation ($\alpha > 1$) if lethality occurs after age x_{s}:

$$x > x_{\mathrm{s}} \tag{17}$$

with

$$\boxed{x_{\mathrm{s}} = \frac{1}{\mu} \ln \left(\frac{\alpha}{\alpha - 1} \right)} \tag{18}$$

Notably, we have $\frac{\alpha}{\alpha-1} > 1$, hence $x_{\mathrm{s}} > 0$.

Drift favors the invasion of the mutation

The mutation is deleterious when $s(x, \alpha) \leq 0$, which occurs for:

$$\begin{cases} \alpha \leq 1 \\ \alpha > 1 \text{ and } x \leq x_{\mathrm{s}} \end{cases} \tag{19}$$

When the mutation is deleterious, genetic drift can favor the invasion of the mutation when $|s(x,\alpha)| < 1/N_\mathrm{e}$, with N_e the effective population size. Here, this inequality is equivalent to:

$$1 - \alpha\left(1 - e^{-\mu x}\right) < \frac{1}{N_\mathrm{e}} \tag{20}$$

$$\alpha\left(1 - e^{-\mu x}\right) > 1 - \frac{1}{N_\mathrm{e}} \tag{21}$$

This means that a mutation cannot invade if $\alpha \leq 1 - \frac{1}{N_\mathrm{e}}$, i.e., if the fecundity cost is too strong.

For $\alpha > 1 - \frac{1}{N_\mathrm{e}}$, the age $\hat{x}$ from which a deleterious mutation can spread (drift barrier) is found by solving:

$$|s(\hat{x},\alpha)| = 1 - \alpha\left(1 - e^{-\mu\hat{x}}\right) = \frac{1}{N_\mathrm{e}} \tag{22}$$

Therefore:

$$\boxed{\hat{x} = \frac{1}{\mu}\ln\left(\frac{\alpha}{N_\mathrm{e}(\alpha-1)+1} \times N_\mathrm{e}\right)} \tag{23}$$

This drift barrier is defined for $\alpha > 1 - \frac{1}{N_\mathrm{e}}$. (otherwise, $\ln\left(\frac{\alpha N_\mathrm{e}}{N_\mathrm{e}(\alpha-1)+1}\right)$ is not defined). Under this condition, we have $\frac{\alpha N_\mathrm{e}}{N_\mathrm{e}(\alpha-1)+1} \geq 1$, and therefore $\hat{x} \geq 0$.
Notably, we get for $\alpha = 1$

$$\hat{x} = \frac{\ln(N_\mathrm{e})}{\mu} \tag{24}$$

Just like in the case with a lethal mutation with no effect of fecundity (Lehtonen 2020).

To compare $\hat{x}$ and x_s, we calculate:

$$\hat{x} - x_\mathrm{s} = \frac{1}{\mu}\left[\ln\left(\frac{\alpha N_\mathrm{e}}{N_\mathrm{e}(\alpha-1)+1}\right) - \ln\left(\frac{\alpha}{\alpha-1}\right)\right] \tag{25}$$

$$\hat{x} - x_\mathrm{s} = \frac{1}{\mu}\ln\left(\frac{\alpha N_\mathrm{e}(\alpha-1)}{\alpha N_\mathrm{e}(\alpha-1)+\alpha}\right) < 0 \tag{26}$$

Hence,

$$\boxed{\hat{x} < x_\mathrm{s}} \tag{27}$$

To compare the drift barrier to the one obtained assuming no effect of fecundity ($\frac{\ln(N_\mathrm{e})}{\mu}$; Lehtonen 2020), we calculate:

$$\frac{\alpha}{N_\mathrm{e}(\alpha-1)+1} - 1 = \frac{-(N_\mathrm{e}-1)(\alpha-1)}{N_\mathrm{e}(\alpha-1)+1} \tag{28}$$

If $N_\mathrm{e} > 1$, we have $\frac{\alpha}{N_\mathrm{e}(\alpha-1)+1} - 1 > 0$, and therefore $\hat{x} > \frac{\ln(N_\mathrm{e})}{\mu}$, for $\alpha \in [1 - \frac{1}{N_\mathrm{e}}, 1]$ (numerator and denumerator of Equation 28 are both > 0).
For a pleiotropic mutation ($\alpha > 1$), selection purging the lethal mutation is getting weaker. As a result, drift can favor the invasion of such lethal mutations at earlier ages; hence, $\hat{x} < \frac{\ln(N_\mathrm{e})}{\mu}$.
For a lethal mutation incurring a fecundity cost ($\alpha \in [1 - \frac{1}{N_\mathrm{e}}, 1]$), selection purging the lethal mutation is getting stronger. As a result, drift can favor the invasion of such lethal mutations at later ages; hence, $\hat{x} > \frac{\ln(N_\mathrm{e})}{\mu}$.
Remember that for $\alpha < 1 - \frac{1}{N_\mathrm{e}}$, no mutations can invade, even those that are lethal very late in life.

More generally, we have:

$$\frac{\partial|s(x,\alpha)|}{\partial\alpha} = -\left(1 - e^{-\mu\, x}\right) < 0 \tag{29}$$

$$\frac{\partial \hat{x}}{\partial \alpha} = \frac{-N_\mathrm{e}\left(N_\mathrm{e}-1\right)}{\mu\alpha N_\mathrm{e}\left(N_\mathrm{e}\left(\alpha-1\right)+1\right)} < 0 \tag{30}$$

Given that $\alpha > 1 - \frac{1}{N_\mathrm{e}}$ and $N_\mathrm{e} > 1$.
Therefore, as the fecundity associated with the lethal mutation increases (increased α), selection purging the lethal mutation is getting weaker, i.e., $\frac{\partial |s(x,\alpha)|}{\partial \alpha} < 0$. Drift can favor the invasion of such lethal mutations at earlier ages; hence, $\frac{\partial \hat{x}}{\partial \alpha} < 0$.

Summary of the sensitivity of the model to parameters

A mutation incurring a very strong fecundity cost ($\alpha \leq 1 - \frac{1}{N_\mathrm{e}}$) cannot invade at all.

A lethal mutation incurring a weak fecundity cost ($1 - \frac{1}{N_\mathrm{e}} < \alpha \leq 1$) can invade via genetic drift for $x > \hat{x}$. The drift barrier $\hat{x}$ decreases as α increases.

A lethal mutation incurring a fecundity benefit (i.e., pleiotropic; $\alpha > 1$) can invade via genetic drift for $\hat{x} < x < x_\mathrm{s}$. It can invade via positive selection for $x \geq x_\mathrm{s}$. The drift barrier $\hat{x}$ decreases as α increases.

A3. Conditions of invasion with a lethal mutation fixed in the population

We assume now that there is a lethal mutation fixed in the population. This lethal mutation is expressed at age $X > 0$ (typically, one can assume that $X = \frac{\ln(N_e)}{\mu}$).
A stationary population implies a lifetime reproductive success equals to one. Here, life ends at age X:

$$\mathrm{LRS} = \int_0^X F.\, e^{-\mu t}\, \mathrm{d}t = 1 \tag{31}$$

Thus:

$$F = \frac{1}{\int_0^X e^{-\mu t}\, \mathrm{d}t} \tag{32}$$

Therefore, in the case with a lethal mutation fixed in the population, we have:

$$F = \frac{\mu}{1 - e^{-\mu\, X}} \tag{33}$$

The fecundity is therefore higher than in the case without any deleterious mutation fixed in the population ($F > \mu$).

Then, the lifetime reproductive success of a mutant expressing a **new** lethal mutation at age x, such that $x \leq X$, and having a different fecundity $= \alpha\, F$ with $\alpha \in [0, +\infty[$, is:

$$\mathrm{LRS}_{\mathrm{mut}} = \int_0^x \alpha\, F.\, l(t)\, \mathrm{d}t \tag{34}$$

We have $F = \frac{\mu}{1-e^{-\mu\, X}}$, and therefore:

$$\mathrm{LRS}_{\mathrm{mut}} = \alpha \int_0^x \frac{\mu}{1 - e^{-\mu\, X}}\, e^{-\mu t}\, \mathrm{d}t \tag{35}$$

$$\mathrm{LRS}_{\mathrm{mut}} = \frac{\alpha}{1 - e^{-\mu\, X}} \int_0^x \mu.\, e^{-\mu t}\, \mathrm{d}t = \frac{\alpha}{1 - e^{-\mu\, X}} \left[-e^{-\mu t}\right]_0^x = \alpha\, \frac{1 - e^{-\mu\, x}}{1 - e^{-\mu\, X}} \tag{36}$$

The selection coefficient $s(x, \alpha, X)$ against such lethal mutation expressed at age x, and affecting fecundity by a factor α is expressed as:

$$s(x, \alpha, X) = \mathrm{LRS}_{\mathrm{mut}} - \mathrm{LRS} \tag{37}$$

And here:

$$s(x, \alpha, X) = \alpha\, \frac{1 - e^{-\mu\, x}}{1 - e^{-\mu\, X}} - 1 \tag{38}$$

Selection can either favor or inhibit the invasion of this mutation, i.e., $s(x, \alpha, X)$ can be positive or negative.

Selection favors the invasion of the mutation

Selection favors the invasion of this mutation when $s(x, \alpha, X) > 0$, which occurs when:

$$\alpha\, \frac{1 - e^{-\mu\, x}}{1 - e^{-\mu\, X}} - 1 > 0 \tag{39}$$

$$\alpha\, \frac{1 - e^{-\mu\, x}}{1 - e^{-\mu\, X}} > 1 \tag{40}$$

Given that $x < X$, we have $\frac{1-e^{-\mu x}}{1-e^{-\mu X}} \leq 1$. Therefore, there are x values leading to $s(x, \alpha, X) > 0$ only for $\alpha > 1$.

$$x > \frac{1}{\mu} \ln\left(\frac{\alpha}{\alpha - 1 + e^{-\mu X}}\right) \tag{41}$$

We have $\frac{\alpha}{\alpha-1+e^{-\mu X}} > 0$ for $\alpha > 1$, hence $\ln\left(\frac{\alpha}{\alpha-1+e^{-\mu X}}\right)$ is defined.
Thus, selection can favor the invasion of a pleiotropic mutation ($\alpha \geq 1$) if lethality occurs after age x_{s}:

$$x > x_{\mathrm{s}} \tag{42}$$

with

$$\boxed{x_{\mathrm{s}} = \frac{1}{\mu} \ln\left(\frac{\alpha}{\alpha - 1 + e^{-\mu X}}\right)} \tag{43}$$

Notably, we have $\frac{\alpha}{\alpha-1+e^{-\mu X}} > 1$, hence $x_{\mathrm{s}} > 0$.

Drift favors the invasion of the mutation

The mutation is deleterious when $s(x, \alpha, X) \leq 0$, which occurs for:

$$\begin{cases} \alpha \leq 1 \\ \alpha > 1 \text{ and } x \leq x_{\mathrm{s}} \end{cases} \tag{44}$$

When the mutation is deleterious, genetic drift can favor the invasion of the mutation when $|s(x, \alpha, X)| < 1/N_{\mathrm{e}}$, with N_{e} the effective population size. Here, this inequality is equivalent to:

$$1 - \alpha \frac{1 - e^{-\mu x}}{1 - e^{-\mu X}} < \frac{1}{N_{\mathrm{e}}} \tag{45}$$

$$\alpha \frac{1 - e^{-\mu x}}{1 - e^{-\mu X}} > 1 - \frac{1}{N_{\mathrm{e}}} \tag{46}$$

Given that $x < X$, we have $\frac{1-e^{-\mu x}}{1-e^{-\mu X}} < 1$. Therefore:

$$\alpha \frac{1 - e^{-\mu x}}{1 - e^{-\mu X}} < \alpha \tag{47}$$

Hence, the drift barrier exists if $\alpha > 1 - \frac{1}{N_{\mathrm{e}}}$.
This means that a mutation cannot invade if $\alpha \leq 1 - \frac{1}{N_{\mathrm{e}}}$, i.e., if the fecundity cost is too strong.

For $\alpha > 1 - \frac{1}{N_{\mathrm{e}}}$, the age $\hat{x}$ from which a deleterious mutation can spread (drift barrier) is found by solving:

$$|s(\hat{x}, \alpha)| = 1 - \alpha \frac{1 - e^{-\mu \hat{x}}}{1 - e^{-\mu X}} = \frac{1}{N_{\mathrm{e}}} \tag{48}$$

Therefore:

$$\boxed{\hat{x} = \frac{1}{\mu} \ln\left(\frac{\alpha}{N_{\mathrm{e}}(\alpha - 1) + 1 + e^{-\mu X}(N_{\mathrm{e}} - 1)} \times N_{\mathrm{e}}\right)} \tag{49}$$

We have $\frac{\alpha N_{\mathrm{e}}}{N_{\mathrm{e}}(\alpha-1)+1+e^{-\mu X}(N_{\mathrm{e}}-1)} > 0$ for $\alpha > \left(1 - \frac{1}{N_{\mathrm{e}}}\right)\left(1 - e^{-\mu X}\right)$ and therefore for $\alpha > 1 - \frac{1}{N_{\mathrm{e}}}$, hence $\ln\left(\frac{\alpha N_{\mathrm{e}}}{N_{\mathrm{e}}(\alpha-1)+1+e^{-\mu X}(N_{\mathrm{e}}-1)}\right)$ is defined. Additionally, we have $\frac{\alpha N_{\mathrm{e}}}{N_{\mathrm{e}}(\alpha-1)+1+e^{-\mu X}(N_{\mathrm{e}}-1)} \geq 1$ because $(N_{\mathrm{e}} - 1)(1 - e^{-\mu X}) \geq 0$, therefore $\hat{x} \geq 0$.

Notably, we get:

$$\hat{x} \xrightarrow[X\to+\infty]{} \frac{1}{\mu}\ln\left(\frac{\alpha}{N_{\mathrm{e}}(\alpha-1)+1}\times N_{\mathrm{e}}\right) \tag{50}$$

Just like in the case without a lethal mutation fixed in the population (see previous section).

To compare $\hat{x}$ and x_{s}, we calculate:

$$\hat{x}-x_{\mathrm{s}} = \frac{1}{\mu}\left[\ln\left(\frac{\alpha N_{\mathrm{e}}}{N_{\mathrm{e}}(\alpha-1)+1+e^{-\mu X}\,(N_{\mathrm{e}}-1)}\right)-\ln\left(\frac{\alpha}{\alpha-1+e^{-\mu X}}\right)\right] \tag{51}$$

$$\hat{x}-x_{\mathrm{s}} = \frac{1}{\mu}\ln\left(\frac{\alpha N_{\mathrm{e}}(\alpha-1+e^{-\mu X})}{\alpha N_{\mathrm{e}}(\alpha-1+e^{-\mu X})+\alpha\,(1-e^{-\mu X})}\right) < 0 \tag{52}$$

Hence,

$$\boxed{\hat{x} < x_{\mathrm{s}}} \tag{53}$$

To compare the drift barrier to the one obtained assuming no lethal mutation fixed in the population (see previous section), we calculate:

$$\frac{\alpha}{N_{\mathrm{e}}(\alpha-1)+1+e^{-\mu X}\,(N_{\mathrm{e}}-1)} - \frac{\alpha}{N_{\mathrm{e}}(\alpha-1)+1} = \frac{-\alpha\,(N_{\mathrm{e}}-1)\,e^{-\mu X}}{\left(N_{\mathrm{e}}(\alpha-1)+1+e^{-\mu X}\,(N_{\mathrm{e}}-1)\right)\left(N_{\mathrm{e}}(\alpha-1)+1\right)} < 0 \tag{54}$$

The fixation of a lethal mutation in the population (ending life at age X) weakens selection purging any other lethal mutations even those having a pleitropic effect, leading to a decrease of the drift barrier associated with those mutations.

More generally, we have:

$$\frac{\partial |s(x,\alpha,X)|}{\partial(-X)} = \frac{-\alpha\,(1-e^{-\mu x})\,e^{-\mu X}}{(1-e^{-\mu X})^2} < 0 \tag{55}$$

$$\frac{\partial \hat{x}}{\partial(-X)} = \frac{-\,(N_{\mathrm{e}}-1)\,e^{-\mu X}}{N_{\mathrm{e}}(\alpha-1)+1+e^{-\mu X}\,(N_{\mathrm{e}}-1)} < 0 \tag{56}$$

Given that $\alpha > 1-\frac{1}{N_{\mathrm{e}}}$ and $N_{\mathrm{e}} > 1$.
Therefore, as all individuals die ealier (decreased X), selection purging the lethal mutation is getting weaker, i.e., $\frac{\partial|s(x,\alpha,X)|}{\partial(-X)} < 0$. Drift can favor the invasion of such lethal mutations at later ages; hence, $\frac{\partial\hat{x}}{\partial(-X)} < 0$.

Summary of the sensitivity of the model to parameters

The fecundity of the mutant has the same effect as in the simple model:
A mutation incurring a very strong fecundity cost ($\alpha \leq 1-\frac{1}{N_{\mathrm{e}}}$) cannot invade at all.

A lethal mutation incurring a weak fecundity cost ($1-\frac{1}{N_{\mathrm{e}}} < \alpha \leq 1$) can invade via genetic drift for $x > \hat{x}$. The drift barrier $\hat{x}$ decreases as α increases.

A lethal mutation incurring a fecundity benefit (i.e., pleiotropic; $\alpha > 1$) can invade via genetic drift for $\hat{x} < x < x_{\mathrm{s}}$. It can invade via positive selection for $x \geq x_{\mathrm{s}}$. The drift barrier $\hat{x}$ decreases as α increases.

The fixation of a lethal mutation leads to a decrease of the drift barrier:
The fixation of a lethal mutation in the population (ending life at age X) weakens selection purging any other lethal mutations, even those that are pleiotropic ($\alpha > 1$), leading to a decrease of the drift barrier associated with those mutations. In other words, the drift barrier $\hat{x}$ decreases as X decreases.

Comparison between x̂ and X

Equation 48 is equivalent to:

$$\frac{1-e^{-\mu\,\hat{x}}}{1-e^{-\mu\,X}}=\frac{1}{\alpha}\left(1-\frac{1}{N_{\mathrm{e}}}\right) \tag{57}$$

Given the conditions of existence of the drift barrier, $\alpha > 1-\frac{1}{N_{\mathrm{e}}}$, we have $\frac{1}{\alpha}\left(1-\frac{1}{N_{\mathrm{e}}}\right)<1$. Therefore:

$$1-e^{-\mu\,\hat{x}}<1-e^{-\mu\,X} \tag{58}$$

$$\hat{x}<X \tag{59}$$

Long-term evolutionary outcome when mutations can be expressed at any age

After lethal mutations invading subsequently, the maximum life span will correspond to the drift barrier below which no lethal mutation can invade. For $\hat{x}=X=X^*$, with X^* being the maximum life span at evolutionary equilibrium, we get from Equation 48:

$$\left(1-e^{-\mu\,X^*}\right)\left(N_{\mathrm{e}}\left(\alpha-1\right)+1\right)=0 \tag{60}$$

Yet, the condition of existence of the drift barrier is $\alpha > 1-\frac{1}{N_{\mathrm{e}}}$, which is equivalent to $N_{\mathrm{e}}\left(\alpha-1\right)+1>0$. Therefore, we get $\hat{x}=X=X^*$ for:

$$1-e^{-\mu\,X^*}=0 \tag{61}$$

$$\boxed{X^*\to 0} \tag{62}$$

Lethal mutations will keep invading and will be expressed at earlier ages, until age $X^*\to 0$.
This is confirmed by the expression of the value of $\hat{x}$ when X approaches 0:

$$\lim_{X\to 0}\hat{x}=0 \tag{63}$$

Long-term evolutionary outcome depending on the grain of age-dependent mutation expression

Let assume that lethal mutations can be expressed every δ time steps. Parameter δ therefore describes the grain of age-dependent mutation expression.

Then the last lethal mutation that may be able to invade is the one expressed at age $\hat{x}$ when $X^\dagger-\hat{x}=\delta$, i.e. at the drift barrier assuming a lethal mutation expressed at age $X^\dagger$ is already fixed. In that case, the expected maximum life span is $X^*_\delta=X^\dagger-\delta$, given that this last mutation is expected to invade.

We express $X^\dagger$ from $X^\dagger-\hat{x}=\delta$:

$$X^\dagger-\frac{1}{\mu}\ln\left(\frac{\alpha}{N_{\mathrm{e}}(\alpha-1)+1+e^{-\mu\,X^\dagger}\left(N_{\mathrm{e}}-1\right)}\times N_{\mathrm{e}}\right)=\delta \tag{64}$$

This leads to:

$$X^\dagger=\frac{1}{\mu}\ln\left(\frac{N_{\mathrm{e}}\left(\alpha\,e^{\mu\,\delta}-1\right)+1}{N_{\mathrm{e}}\left(\alpha-1\right)+1}\right) \tag{65}$$

Therefore, the expected maximum life span X^*_δ under a grain of age-dependence δ is:

$$X^*_\delta=\frac{1}{\mu}\ln\left(\frac{N_{\mathrm{e}}\left(\alpha\,e^{\mu\,\delta}-1\right)+1}{N_{\mathrm{e}}\left(\alpha-1\right)+1}\right)-\delta \tag{66}$$

$$\boxed{X_\delta^* = \frac{1}{\mu} \ln \left(\frac{\alpha\, N_\mathrm{e} - e^{-\mu\, \delta}\, (N_\mathrm{e} - 1)}{N_\mathrm{e}\, (\alpha - 1) + 1} \right)} \tag{67}$$

Notably, without pleiotropy ($\alpha = 1$), we get: $X_\delta^* = \frac{1}{\mu} \ln \left(N_\mathrm{e} - e^{-\mu\, \delta}\, (N_\mathrm{e} - 1) \right)$. This is analog to Lehtonen's (2020) expression of the drift barrier, except that a fine grain of age-dependence (low δ) lowers the expected maximum life span.

Indeed, we have:

$$\frac{\partial X_\delta^*}{\partial \delta} = \frac{N_\mathrm{e} - 1}{N_\mathrm{e}\, (\alpha - 1) + 1} > 0 \tag{68}$$

Therefore, the expected maximum life span increases with the grain of age-dependence of mutation expression.

Additionally, we get:

$$\lim_{\delta \to \infty} X_\delta^* = \frac{1}{\mu} \ln \left(\frac{\alpha\, N_\mathrm{e}}{N_\mathrm{e}(\alpha - 1) + 1} \right) \tag{69}$$

Which is the expression of the drift barrier when accounting for pleiotropy. Without pleiotropy ($\alpha = 1$), we get:

$$\lim_{\delta \to \infty} X_\delta^* = \frac{1}{\mu} \ln \left(N_\mathrm{e} \right) \tag{70}$$

We also get:

$$\lim_{\delta \to 0} X_\delta^* = 0 \tag{71}$$

Which corresponds to the expected maximum life span when mutations can be expressed at any age.

A4. Conditions of invasion with a lethal mutation fixed in the population when recruitment is limiting

We assume now that there is a lethal mutation fixed in the population. This lethal mutation is expressed at age X, below the age at which fecundity X_{F} compensates the increase in mortality, i.e. $X \leq X_{\mathrm{F}}$.
Here, life ends at age X:

$$\mathrm{LRS} = \int_0^X F.\, e^{-\mu t}\,\mathrm{d}t < 1 \tag{72}$$

With:

$$F = \frac{1}{\int_0^{X_{\mathrm{F}}} e^{-\mu t}\,\mathrm{d}t} \tag{73}$$

Therefore, in the case with a lethal mutation fixed in the population, we have:

$$F = \frac{\mu}{1 - e^{-\mu\, X_{\mathrm{F}}}} \tag{74}$$

Then, the lifetime reproductive success of a mutant expressing a **new** lethal mutation at age x, such that $x \leq X$, and having a different fecundity $= \alpha\, F$ with $\alpha \in [0, +\infty[$, is:

$$\mathrm{LRS}_{\mathrm{mut}} = \int_0^x \alpha\, F.\, l(t)\,\mathrm{d}t \tag{75}$$

We have $F = \frac{\mu}{1-e^{-\mu\, X_{\mathrm{F}}}}$, and therefore:

$$\mathrm{LRS}_{\mathrm{mut}} = \alpha \int_0^x \frac{\mu}{1 - e^{-\mu\, X_{\mathrm{F}}}}\, e^{-\mu t}\,\mathrm{d}t \tag{76}$$

$$\mathrm{LRS}_{\mathrm{mut}} = \frac{\alpha}{1 - e^{-\mu\, X_{\mathrm{F}}}} \int_0^x \mu.\, e^{-\mu t}\,\mathrm{d}t = \frac{\alpha}{1 - e^{-\mu\, X_{\mathrm{F}}}} \left[-e^{-\mu t}\right]_0^x = \alpha\, \frac{1 - e^{-\mu\, x}}{1 - e^{-\mu\, X_{\mathrm{F}}}} \tag{77}$$

The selection coefficient $s(x, \alpha, X)$ against such lethal mutation expressed at age x, and affecting fecundity by a factor α is expressed as:

$$s(x, \alpha, X) = \mathrm{LRS}_{\mathrm{mut}} - \mathrm{LRS} \tag{78}$$

And here:

$$s(x, \alpha, X) = \alpha\, \frac{1 - e^{-\mu\, x}}{1 - e^{-\mu\, X_{\mathrm{F}}}} - 1 \tag{79}$$

Selection can either favor or inhibit the invasion of this mutation, i.e., $s(x, \alpha, X)$ can be positive or negative.

Selection favors the invasion of the mutation

Selection favors the invasion of this mutation when $s(x, \alpha, X) > 0$, which occurs when:

$$\alpha\, \frac{1 - e^{-\mu\, x}}{1 - e^{-\mu\, X_{\mathrm{F}}}} - 1 > 0 \tag{80}$$

$$\alpha\, \frac{1 - e^{-\mu\, x}}{1 - e^{-\mu\, X_{\mathrm{F}}}} > 1 \tag{81}$$

Given that $x < X \leq X_{\mathrm{F}}$, we have $\frac{1-e^{-\mu\, x}}{1-e^{-\mu\, X_{\mathrm{F}}}} \leq 1$. Therefore, there are x values leading to $s(x, \alpha, X) > 0$ only for $\alpha > 1$.

$$x > \frac{1}{\mu} \ln\left(\frac{\alpha}{\alpha - 1 + e^{-\mu\, X_{\mathrm{F}}}}\right) \tag{82}$$

We have $\frac{\alpha}{\alpha-1+e^{-\mu\, X}} > 0$ for $\alpha > 1$, hence $\ln\left(\frac{\alpha}{\alpha-1+e^{-\mu\, X_{\mathrm{F}}}}\right)$ is defined.
Thus, selection can favor the invasion of a pleiotropic mutation ($\alpha \geq 1$) if lethality occurs after age x_{s}:

$$x > x_{\mathrm{s}} \tag{83}$$

with

$$\boxed{x_{\mathrm{s}} = \frac{1}{\mu} \ln\left(\frac{\alpha}{\alpha - 1 + e^{-\mu\, X_{\mathrm{F}}}}\right)} \tag{84}$$

Notably, we have $\frac{\alpha}{\alpha-1+e^{-\mu\, X_{\mathrm{F}}}} > 1$, hence $x_{\mathrm{s}} > 0$.

Drift favors the invasion of the mutation

The mutation is deleterious when $s(x, \alpha, X) \leq 0$, which occurs for:

$$\begin{cases} \alpha \leq 1 \\ \alpha > 1 \text{ and } x \leq x_{\mathrm{s}} \end{cases} \tag{85}$$

When the mutation is deleterious, genetic drift can favor the invasion of the mutation when $|s(x, \alpha, X)| < 1/N_{\mathrm{e}}$, with N_{e} the effective population size. Here, this inequality is equivalent to:

$$1 - \alpha\, \frac{1 - e^{-\mu\, x}}{1 - e^{-\mu\, X_{\mathrm{F}}}} < \frac{1}{N_{\mathrm{e}}} \tag{86}$$

$$\alpha\, \frac{1 - e^{-\mu\, x}}{1 - e^{-\mu\, X_{\mathrm{F}}}} > 1 - \frac{1}{N_{\mathrm{e}}} \tag{87}$$

Given that $x < X_{\mathrm{F}}$, we have $\frac{1-e^{-\mu\, x}}{1-e^{-\mu\, X_{\mathrm{F}}}} < 1$. Therefore:

$$\alpha\, \frac{1 - e^{-\mu\, x}}{1 - e^{-\mu\, X_{\mathrm{F}}}} < \alpha \tag{88}$$

Hence, the drift barrier exists if $\alpha > 1 - \frac{1}{N_{\mathrm{e}}}$.
This means that a mutation cannot invade if $\alpha \leq 1 - \frac{1}{N_{\mathrm{e}}}$, i.e., if the fecundity cost is too strong.

For $\alpha > 1 - \frac{1}{N_{\mathrm{e}}}$, the age $\hat{x}$ from which a deleterious mutation can spread (drift barrier) is found by solving:

$$|s(\hat{x}, \alpha)| = 1 - \alpha\, \frac{1 - e^{-\mu\, \hat{x}}}{1 - e^{-\mu\, X_{\mathrm{F}}}} = \frac{1}{N_{\mathrm{e}}} \tag{89}$$

Therefore:

$$\boxed{\hat{x} = \frac{1}{\mu} \ln\left(\frac{\alpha}{N_{\mathrm{e}}(\alpha - 1) + 1 + e^{-\mu\, X_{\mathrm{F}}}\,(N_{\mathrm{e}} - 1)} \times N_{\mathrm{e}}\right)} \tag{90}$$

We note that $\hat{x}$ depends on X_{F}, and not on X.
We have $\frac{\alpha N_{\mathrm{e}}}{N_{\mathrm{e}}(\alpha-1)+1+e^{-\mu\, X_{\mathrm{F}}}(N_{\mathrm{e}}-1)} > 0$ for $\alpha > \left(1 - \frac{1}{N_{\mathrm{e}}}\right)\left(1 - e^{-\mu\, X_{\mathrm{F}}}\right)$ and therefore for $\alpha > 1 - \frac{1}{N_{\mathrm{e}}}$, hence $\ln\left(\frac{\alpha N_{\mathrm{e}}}{N_{\mathrm{e}}(\alpha-1)+1+e^{-\mu\, X_{\mathrm{F}}}(N_{\mathrm{e}}-1)}\right)$ is defined. Additionally, we have $\frac{\alpha N_{\mathrm{e}}}{N_{\mathrm{e}}(\alpha-1)+1+e^{-\mu\, X_{\mathrm{F}}}(N_{\mathrm{e}}-1)} \geq 1$ because $(N_{\mathrm{e}} - 1)(1 - e^{-\mu\, X_{\mathrm{F}}}) \geq 0$, therefore $\hat{x} \geq 0$.

To compare $\hat{x}$ and x_{s}, we calculate:

$$\hat{x} - x_{\mathrm{s}} = \frac{1}{\mu}\left[\ln\left(\frac{\alpha N_{\mathrm{e}}}{N_{\mathrm{e}}(\alpha - 1) + 1 + e^{-\mu\, X_{\mathrm{F}}}\,(N_{\mathrm{e}} - 1)}\right) - \ln\left(\frac{\alpha}{\alpha - 1 + e^{-\mu\, X_{\mathrm{F}}}}\right)\right] \tag{91}$$

$$\hat{x} - x_{\mathrm{s}} = \frac{1}{\mu} \ln\left(\frac{\alpha N_{\mathrm{e}}(\alpha - 1 + e^{-\mu\, X_{\mathrm{F}}})}{\alpha N_{\mathrm{e}}(\alpha - 1 + e^{-\mu\, X_{\mathrm{F}}}) + \alpha\,(1 - e^{-\mu\, X_{\mathrm{F}}})}\right) < 0 \tag{92}$$

Hence,

$$\boxed{\hat{x} < x_{\mathrm{s}}} \tag{93}$$

Comparison between $\hat{\mathbf{x}}$ and $\mathbf{X_F}$

Equation 89 is equivalent to:

$$\frac{1 - e^{-\mu\,\hat{x}}}{1 - e^{-\mu\,X_{\mathrm{F}}}} = \frac{1}{\alpha}\left(1 - \frac{1}{N_{\mathrm{e}}}\right) \tag{94}$$

Given the conditions of existence of the drift barrier, $\alpha > 1 - \frac{1}{N_{\mathrm{e}}}$, we have $\frac{1}{\alpha}\left(1 - \frac{1}{N_{\mathrm{e}}}\right) < 1$. Therefore:

$$1 - e^{-\mu\,\hat{x}} < 1 - e^{-\mu\,X_{\mathrm{F}}} \tag{95}$$

$$\hat{x} < X_{\mathrm{F}} \tag{96}$$

Long-term ecological outcome

During the invasion process of a lethal mutation expressed at age x, below the age at which fecundity X_{f} compensates the increase in mortality, the population growth rate is below 1. This will ultimately lead to the extinction of the population. Nonetheless, we note that the accumulation of lethal mutations would not have been able to lead to a lower maximum life span than $\hat{x}$ shown in Equation 90.

A5. Selection purging many deleterious mutations is getting weaker as any other deleterious mutation gets fixed

While we focused on lethal mutations in the previous sections, we can generalize our results to any other deleterious mutations getting fixed in the population.

Without deleterious mutations increasing mortality fixed in the population

Consider an increasing function $\mu : \mathbb{R}^+ \to \mathbb{R}^+$ with $\mu(0) = 0$ and $\lim_{x \to +\infty} \mu(x) = +\infty$, such that the survival probability from age 0 to age x is equal to $e^{-\mu(x)}$ when accounting only for **extrinsic mortality**. For instance, if extrinsic mortality is constant and occurs at a rate $= \mu$ as in previous sections, then $\mu(x) = \mu \times x$.

Consider an increasing function $\nu : \mathbb{R}^+ \to \mathbb{R}^+$ with $\nu(0) = 0$ and $\lim_{x \to +\infty} \nu(x) = +\infty$, such that the survival probability from age 0 to age x is equal to $e^{-\nu(x)}$ when accounting only for the **expression of a deleterious mutation**. For instance, if the expression of the deleterious mutation is lethal at a particular age $\hat{x}$ as in the previous section, then $\nu(x) = 0$ for $x \in [0, \hat{x}]$ and $\nu(x) = +\infty$ for $x \in [\hat{x}, +\infty[$. Here, we de not make any assumption on the exact nature of the deleterious effect of the mutation; our theory applies equally well to a deleterious mutation that has a non-monotonic effect on mortality (e.g., mortality associated with the mutation can increase or decrease as the individual ages). Indeed, whatever the nature of the per-age mortality function, the function ν, such that the survival probability from age 0 to age x is equal to $e^{-\nu(x)}$ will be an increasing function.

A stationary population implies a lifetime reproductive success equals to one. Then, the lifetime reproductive success of an individual that does not express the deleterious mutation is:

$$\text{LRS} = 1 \tag{97}$$

and the fecundity is the same at all ages:

$$F = \frac{1}{\int_{t=0}^{+\infty} e^{-\mu(t)} \, \mathrm{d}t} \tag{98}$$

The lifetime reproductive success of an individual expressing the deleterious mutation that also affects fecundity by a factor $\alpha(x)$ at age x is:

$$\text{LRS}_{\text{mut}} = \frac{\int_{x=0}^{+\infty} \alpha(x) \, e^{-\mu(x)-\nu(x)} \, \mathrm{d}x}{\int_{t=0}^{+\infty} e^{-\mu(t)} \, \mathrm{d}t} \tag{99}$$

And the selection coefficient s against such deleterious mutation is:

$$s = \frac{\int_{x=0}^{+\infty} \alpha(x) \, e^{-\mu(x)-\nu(x)} \, \mathrm{d}x}{\int_{t=0}^{+\infty} e^{-\mu(t)} \, \mathrm{d}t} - 1 \tag{100}$$

$$s = \frac{\int_{x=0}^{+\infty} \alpha(x) \, e^{-\mu(x)-\nu(x)} \, \mathrm{d}x - \int_{x=0}^{+\infty} e^{-\mu(x)} \, \mathrm{d}x}{\int_{t=0}^{+\infty} e^{-\mu(t)} \, \mathrm{d}t} \tag{101}$$

$$s = \frac{\int_{x=0}^{+\infty} \left[\alpha(x) \, e^{-\mu(x)-\nu(x)} - e^{-\mu(x)} \right] \mathrm{d}x}{\int_{t=0}^{+\infty} e^{-\mu(t)} \, \mathrm{d}t} \tag{102}$$

$$s = \frac{\int_{x=0}^{+\infty} e^{-\mu(x)} \left(\alpha(x) \, e^{-\nu(x)} - 1 \right) \mathrm{d}x}{\int_{t=0}^{+\infty} e^{-\mu(t)} \, \mathrm{d}t} \tag{103}$$

With deleterious mutations increasing mortality fixed in the population

Now consider an increasing function $\Delta_\mu : \mathbb{R}^+ \to \mathbb{R}^+$ with $\Delta_\mu(0) = 0$ and $\lim_{x\to+\infty} \Delta_\mu(x) = +\infty$, such that the survival probability from age 0 to age x is equal to $e^{-\Delta_\mu(x)}$ when accounting only for the **expression of a deleterious mutation that is fixed in the population**. For instance, if the expression of the fixed deleterious mutation is lethal at a particular age X as in the previous section, then $\Delta_\mu(x) = 0$ for $x \in [0, X]$ and $\Delta_\mu(x) = +\infty$ for $x \in [X, +\infty[$.

A stationary population implies a lifetime reproductive success equals to one. Then, the lifetime reproductive success of an individual that does not express the deleterious mutation is:

$$\mathrm{LRS} = 1 \tag{104}$$

and the fecundity is the same at all ages:

$$F = \frac{1}{\int_{t=0}^{+\infty} e^{-\mu(t)-\Delta_\mu(t)}\,\mathrm{d}t} \tag{105}$$

The lifetime reproductive success of an individual expressing the deleterious mutation that also affects fecundity by a factor $\alpha(x)$ at age x is:

$$\mathrm{LRS_{mut}} = \frac{\int_{x=0}^{+\infty} \alpha(x)\, e^{-\mu(x)-\Delta_\mu(x)-\nu(x)}\,\mathrm{d}x}{\int_{t=0}^{+\infty} e^{-\mu(t)-\Delta_\mu(t)}\,\mathrm{d}t} \tag{106}$$

And the selection coefficient s against such deleterious mutation is:

$$s_\Delta = \frac{\int_{x=0}^{+\infty} e^{-\mu(x)-\Delta_\mu(x)} \left(\alpha(x)\, e^{-\nu(x)} - 1\right)\,\mathrm{d}x}{\int_{t=0}^{+\infty} e^{-\mu(t)-\Delta_\mu(t)}\,\mathrm{d}t} \tag{107}$$

Comparison of the selection coefficients with/without deleterious mutations increasing mortality fixed in the population

Let us consider that the function α satisfies the conditions under which the selection coefficients are negative; i.e., $s < 0$ and $s_\Delta < 0$.
Then the difference of strength of selection with/without deleterious mutations increasing mortality fixed in the population is:

$$|s_\Delta| - |s| = \frac{-\int_{x=0}^{+\infty} \alpha(x)\, e^{-\mu(x)-\Delta_\mu(x)-\nu(x)}\,\mathrm{d}x}{\int_{t=0}^{+\infty} e^{-\mu(t)-\Delta_\mu(t)}\,\mathrm{d}t} + \frac{\int_{x=0}^{+\infty} \alpha(x)\, e^{-\mu(x)-\nu(x)}\,\mathrm{d}x}{\int_{t=0}^{+\infty} e^{-\mu(t)}\,\mathrm{d}t} \tag{108}$$

$$|s_\Delta| - |s| = \frac{-\int_{x=0}^{+\infty} \alpha(x) e^{-\mu(x)-\Delta_\mu(x)-\nu(x)}\,\mathrm{d}x \times \int_{t=0}^{+\infty} e^{-\mu(t)}\,\mathrm{d}t + \int_{x=0}^{+\infty} \alpha(x) e^{-\mu(x)-\nu(x)}\,\mathrm{d}x \times \int_{t=0}^{+\infty} e^{-\mu(t)-\Delta_\mu(t)}\,\mathrm{d}t}{\int_{t=0}^{+\infty} e^{-\mu(t)-\Delta_\mu(t)}\,\mathrm{d}t \times \int_{t=0}^{+\infty} e^{-\mu(t)}\,\mathrm{d}t} \tag{109}$$

$$|s_\Delta| - |s| = \frac{-\int_{x=0}^{+\infty}\int_{t=0}^{+\infty} \alpha(x) e^{-\mu(x)-\Delta_\mu(x)-\nu(x)-\mu(t)}\,\mathrm{d}x\,\mathrm{d}t + \int_{x=0}^{+\infty}\int_{t=0}^{+\infty} \alpha(x) e^{-\mu(x)-\nu(x)-\mu(t)-\Delta_\mu(t)}\,\mathrm{d}x\,\mathrm{d}t}{\int_{t=0}^{+\infty} e^{-\mu(t)-\Delta_\mu(t)}\,\mathrm{d}t \times \int_{t=0}^{+\infty} e^{-\mu(t)}\,\mathrm{d}t} \tag{110}$$

$$|s_\Delta| - |s| = \frac{\int_{x=0}^{+\infty}\int_{t=0}^{+\infty} \alpha(x) e^{-\mu(x)-\mu(t)-\nu(x)} \left(e^{-\Delta_\mu(t)} - e^{-\Delta_\mu(x)}\right)\,\mathrm{d}x\,\mathrm{d}t}{\int_{t=0}^{+\infty} e^{-\mu(t)-\Delta_\mu(t)}\,\mathrm{d}t \times \int_{t=0}^{+\infty} e^{-\mu(t)}\,\mathrm{d}t} \tag{111}$$

Therefore, $|s_\Delta| - |s|$ can be expressed as:

$$\frac{\int_{t=0}^{+\infty} e^{-\mu(t)} \left[\int_{x=0}^{t} \left(e^{-\Delta_\mu(t)} - e^{-\Delta_\mu(x)}\right) \alpha(x)\, e^{-\mu(x)-\nu(x)}\, \mathrm{d}x + \int_{x=t}^{+\infty} \left(e^{-\Delta_\mu(t)} - e^{-\Delta_\mu(x)}\right) \alpha(x)\, e^{-\mu(x)-\nu(x)}\, \mathrm{d}x\right] \mathrm{d}t}{\int_{t=0}^{+\infty} e^{-\mu(t)-\Delta_\mu(t)}\, \mathrm{d}t \times \int_{t=0}^{+\infty} e^{-\mu(t)} \mathrm{d}t} \tag{112}$$

By essence, Δ_μ is an increasing function. If $x < t$, we get $e^{-\Delta_\mu(t)} - e^{-\Delta_\mu(x)} < 0$. Within the integrale in the numerator, the first integrale ($\int_{x=0}^{t} ... \mathrm{d}x$) is necessarily negative. Similarly, we can conclude that the second integrale ($\int_{x=t}^{+\infty} ... \mathrm{d}x$) is necessarily positive.
The second integrale accounting for $x > t$ is closer to 0 than the first integrale accounting for $x < t$ as soon as $\alpha(x)\, e^{-\mu(x)-\nu(x)}$ is a decreasing function. In that case, the first integrale determines the sign of $|s_\Delta| - |s|$, and we get $|s_\Delta| - |s| \leq 0$, i.e., $|s_\Delta| \leq |s|$.
Notably, $e^{-\mu(x)-\nu(x)}$ is a decreasing function, such that $e^{-\mu(0)-\nu(0)} = 1$ and $\lim_{x\to\infty} e^{-\mu(x)-\nu(x)} = 0$. Therefore, we get $|s_\Delta| \leq |s|$ for all non-pleiotropic deleterious mutation (when $\alpha(x) = 1$ for all x), but also for deleterious mutations with a pleiotropic effect characterized by a decreasing function α, i.e., for mutations increasing fecundity especially at young age. Note however that selection acting on a deleterious mutations with a pleiotropic effect characterized by an increasing function α, i.e., on mutations increasing fecundity especially at old age, can still be characterized by $|s_\Delta| \leq |s|$, if the composite function $\alpha(x)\, e^{-\mu(x)-\nu(x)}$ is decreasing.
Overall, for all non-pleiotropic deleterious mutation, and for most pleiotropic deleterious mutation, we get:

$$\boxed{|s_\Delta| \leq |s|} \tag{113}$$

Therefore, the fixation of any deleterious mutation (reducing survival via the function Δ_μ) is weakening selection purging any non-pleiotropic deleterious mutations (reducing survival via the function ν) and most pleiotropic deleterious mutations (reducing survival via the function ν, and increasing fecundity via the function α). As a result, genetic drift easily favors the invasion of deleterious mutations as soon as other deleterious mutations get fixed.

Our main finding that a lethal mutation can easily invade when another lethal mutation is already fixed in the population can therefore be generalized to non-pleiotropic non-lethal deleterious mutations, but also to most pleiotropic non-lethal deleterious mutations. Here, we did not make any assumption on the exact nature of the deleterious effect of the mutation; and our theory can be well applied to a deleterious mutation that has a non-monotonic effect on mortality.

Appendix B: Individual-based model

B1. The model

Purpose

We investigate how the accumulation of lethal or sub-lethal mutations shapes senescence. In the mathematical models analyzed in Appendix A in Supplementary Material, (1) a threshold value is used to infer whether deleterious mutations can reach fixation or not, (2) populations do not decline or get extinct due to the accumulation of lethal mutations, (3) lethal mutation can be expressed at any age, and (4) there is no reverse mutations that can remove the deleterious effect of lethal mutations. We build here an individual-based model to relax these assumptions that could conceivably have implications for the evolution of senescence.

In our individual-based model, time is discrete, such that each time step corresponds to a day, and we consider a single asexual population subject to local competition. We simulate the accumulation of lethal mutant alleles, the expression of which is age-dependent. In some simulations, the genes are pleiotropic because lethal alleles also increase the individual fecundity. We also consider in other simulations that mutant alleles are non-lethal or that mutations are dependent on the somatic state.

If selection purging late-expressed lethal mutations is too weak, individuals carrying these mutations do not suffer important fitness costs and these lethal mutations can invade in the population. Nonetheless, the fixation of these lethal mutations may translate into lower growth rates, and potentially into population decline; this may cause population extinction in our simulations. We also investigate the implication of the grain of the age-dependence characterizing mutation expression, by assuming that mutations can be expressed at the onset of either any day, any month or any year. We also consider that any lethal mutant allele can mutate into a non-lethal allele (reverse mutations).

The model is implemented in C++ and the scripts are available in a Zenodo repository (doi: 10.5281/zenodo.8392458; `https://zenodo.org/record/8392458`).

State variables

The model comprises two hierarchical levels: individuals and genes.

We consider an asexual population composed of a finite number of asexual haploid individuals characterized by their age. Each individual carries genes, the alleles of which determine the maximum age at which the individual can live. We thus assume that deleterious mutant alleles are expressed in all mutants, as it would be the case of dominant alleles in diploids. Given that deleterious mutant alleles that are recessive are more likely to invade than those that are dominant, we focus on the least favorable situation for the accumulation of deleterious mutations.

Each gene is expressed at a specific age — i.e., at the onset of either any day, any month or any year depending on the grain of age-dependence implemented in the simulation. The ancestral allele of each gene is neutral, and we assume that each gene can mutate, with the mutant allele decreasing the survival probability by a certain factor when expressed (in particular, by a factor 1 if we assume that mutations are lethal). If genes are pleiotropic, the mutant alleles increases the fecundity of the individual. In some simulations, we also assume that each gene can mutate back to its ancestral non-lethal allele. As detailed in the subsection 'Production of offspring and mutations', we categorize genes according to the age at which they are expressed; for instance, for yearly-expressed genes, genes belong to the categories 'expressed at year 0', 'expressed at year 1', 'expressed at year 2', and so on. To reduce simulation runtime, we do not consider each gene individually. We instead follow separately the fate of all the genes belonging to the each category.

In a supplementary simulation, we assume that the expression of mutations is dependent on the somatic state of the individual. Individuals are then characterized by an additional variable that increase stochastically over time and that reflect their somatic state.

Process overview and scheduling

Within each time step, several phases are processed in the following order: extrinsic mortality, intrinsic mortality (i.e., mortality due to the expression of deleterious mutations), production of offspring (including mutation), and local competition among offspring. These population processes are described in the subsection "Submodels" below.

Design concepts

Stochasticity. – All processes are probabilistic. Some probabilities are defined as parameters of the model (probability of adults dying due to extrinsic mortality, mutation probability), and others are computed from the individual's genes (probability of adults dying due to intrinsic mortality, probability of producing offspring if genes are pleiotropic), or from the composition of the population (probability of offspring dying due to local competition).

Interactions. – Offspring individuals compete for resources before being added to the population, i.e., offspring recruitment is density-dependent. The intensity of competition between offspring depends on the total number of individuals (offspring and surviving adults).

Adaptation. – Individuals' maximum life span determines the fitness of individuals; an early death due to intrinsic mortality translates into a lower number of offspring. Likewise, if genes are pleiotropic, fecundity is a fitness component; an early death associated with a high fecundity may translate into a higher number of offspring. Genetic drift may contribute to the evolution of those traits when selection is too weak.

Emergence. – Two important features emerge from the model. First, senescence occurs once some age-dependent lethal mutations reducing the individuals' maximum life span get fixed in the population. Second, the fixation of lethal mutations can ultimately lead to population decline and extinction.

Observation. – Over the course of a simulation, we record the age at which individuals die, the cause of the death (intrinsic or extrinsic mortality), the proportion of individuals dying at each age due to a lethal mutation assuming they reach that age, the proportion of offspring recruited into the population, and the population size.

Initialization

At initial state, we consider a population at its carrying capacity, and we assume that individuals have no lethal mutation.

Sub-models

Increase in the somatic state. – If we consider that mutations are not age-dependent, but rather somatic state-dependent, the somatic state of each individual is initially set to 0 and is incremented at a rate β per year. We convert this rate into a probability of increasing the individual's somatic state per time step: $P_{\text{inc}} = 1 - \exp(-\beta/365)$. Remember that each time step corresponds to a day, hence the factor 1/365.

Extrinsic mortality. – Each adult individual may die due to extrinsic mortality at a rate μ per year. We convert this rate into a probability of dying due to extrinsic mortality per time step: $P_{\text{ext}} = 1 - \exp(-\mu/365)$. In some analyses, we also considered the case where the extrinsic mortality rate is density-dependent, such that

extrinsic mortality is reduced by a factor equal to the ratio between the population density and the carrying capacity; in the extreme case where the population density is close to zero, extrinsic mortality is also close to zero.

Intrinsic mortality. – Each adult individual may die due to intrinsic mortality if it carries a lethal allele that is expressed at her current age (or current somatic state if we assume that the expression of mutations is dependent on the individual's somatic state). In that case, the probability of dying is equal to one. In simulation with sub-lethal mutations, we assume that the probability of dying is increased by a factor 0.1 due to the expression of each sub-lethal mutation.

Production of offspring and mutations. – For simplicity, we assume that all individuals are asexual. An adult individual that does not carry a lethal mutation may give birth to offspring at a rate b_0 per year (subscript 0 stands for the absence of lethal mutation). We convert this rate into a mean number of offspring produced per time step: $B_0 = 1 - \exp(-b_0/365)$. We here use the probability of producing offspring per time step as an approximation of the mean number of offspring given that the probability of producing offspring per day is typically very low.

If an individual carries lethal mutations, which are expressed at age a_{die} (in days) at the earliest, fecundity may be increased due to pleiotropic effects by a factor $\Gamma = 1+(\alpha_{\max}-1)\, \exp(-\gamma\, a_{\text{die}})$ (see Figure S1). Parameter $\alpha_{\max} \in [1, \infty[$ describes the maximum increase in fecundity due to pleiotropy (for $a_{\text{die}} = 0$). Parameter $\gamma > 0$ describes the rate at which the maximum increase in fecundity is attained as a_{die} decreases. Notably, there is no pleiotropy if $\alpha_{\max} = 1$ (Figure S1). Therefore, the mean number of offspring produced per time step corresponds to $B = \Gamma \times B_0$. Hence, for each surviving adult, a Poisson-distributed number of offspring with mean B is generated.

During the production of offspring, mutation can occur. Each gene is expressed at a given age, and can therefore be categorized depending on the age at which it is expressed. To reduce simulation runtime, we assume that deleterious mutations occur at a rate $m = 2 \times 10^{-3}$ per gene category (instead of tracking the fate of a limited number of genes). Likewise, if the offspring inherits deleterious mutations from her parent, reverse mutations occur at a rate m_{rev} per gene category.

Local competition among offspring. – Offspring survival depends on competition with all other individuals. Survival probability is calculated using an analog of the Beverton-Holt stock-recruitment model (Kot 2001): population growth is logistic as a consequence of density-dependent resource competition. Offspring individuals thus survive to the reproduction stage with probability $v = 1/\left[1 + N_{\text{offspring}}/(K - N_{\text{adult}})\right]$, with $N_{\text{offspring}}$ being the number of offspring, and N_{adult} being the number of adults remaining in the population, following extrinsic and intrinsic mortalities. In contrast to the original Beverton-Holt stock-recruitment model, we assume here that adult do not suffer from local competition, and that offspring compete with each other for the resources that have not been consumed by adults. As a result, the population size after local competition remains around K as long as there are enough offspring to replace the dead adults; otherwise the population declines.

B2. Simulation experiments

Simulations conducted

We conducted simulations with variation in carrying capacity K (reflecting the effective population size), adult mortality rate μ, birth rate b_0, and grain of age-dependence of mutation expression. We also varied the magnitude of pleiotropy ($\alpha_{\max}$), the lethality of deleterious mutations (lethal or sub-lethal mutations), and the existence of 'reverse mutations' that remove the deleterious effect of deleterious mutations.

Each simulation last 100,000 years which is enough time for evolutionary equilibrium to be reached.

Statistics

We record the 97.5th percentile of individual life spans as a proxy of the maximum life span. If population extinction occurs, we record whether it occurs due to extrinsic mortality being too high, or due to the accumulation

of lethal mutations.

B3. Results

We show that in our individual-based model, the accumulation of lethal mutations leads to population extinction if these mutations can be expressed at the onset of each day (left column in Figure S2). As the age-dependency of mutation expression gets coarser, mutation accumulation is more likely to be halted, avoiding population extinction, and the maximum age at death at evolutionary equilibrium gets higher (middle and right column in Figure S2; see Figure S3 showing that the evolutionary equilibrium has been reached and see Figs. S4 and S5 for examples of simulations). In all conditions, however, the maximum age at death is lower than that predicted by Lehtonen (Lehtonen, 2020) when considering the spread of a single lethal mutation (Figure S6).

We also confirm our analytical results by showing that mutation accumulation also occurs when considering pleiotropic mutations, i.e., lethal mutations that increase fecundity at all ages (Figure S7 and S8). As the magnitude of pleiotropy increases (higher $\alpha_{\max}$), population extinction is less likely. The maximum age at death at evolutionary equilibrium does not change considerably.

We also show here that 'reverse' mutations that remove the deleterious effect of lethal mutations have little impact on the dynamics of mutation accumulation (Figure S9). With reverse mutations occurring at the same rate as lethal mutations, population extinction is slightly less likely, and the maximum age at death at evolutionary equilibrium is slightly higher that without reverse mutations. In the case of daily-expressed lethal mutations, mutation accumulation leads to population extinction even with reverse mutations.

We also show that the accumulation of sub-lethal mutations also occurs leading to an even-lower maximum age at death and to an increased likelihood of population extinction (Figure S10). Reverse mutations do not significantly change this result either (Figure S10).

We also considered the case where the extrinsic mortality rate is density-dependent. This does not change qualitatively our results (Figure S11), except that extinction before mutation accumulation requires a lower birth rate and a higher maximum adult mortality rate. The time at which populations collapse is also delayed with such density-dependent adult mortality rate.

Finally, if mutations are expressed depending of the somatic state of the individual that increases at a given rate over time, then the accumulation of lethal mutation is easily stopped if the somatic state changes at a low rate (Figure S12). This occurs for the same reason as when the age-dependency of mutation expression is coarse (e.g., when mutations can be expressed at the onset of every year).

B4. Figures

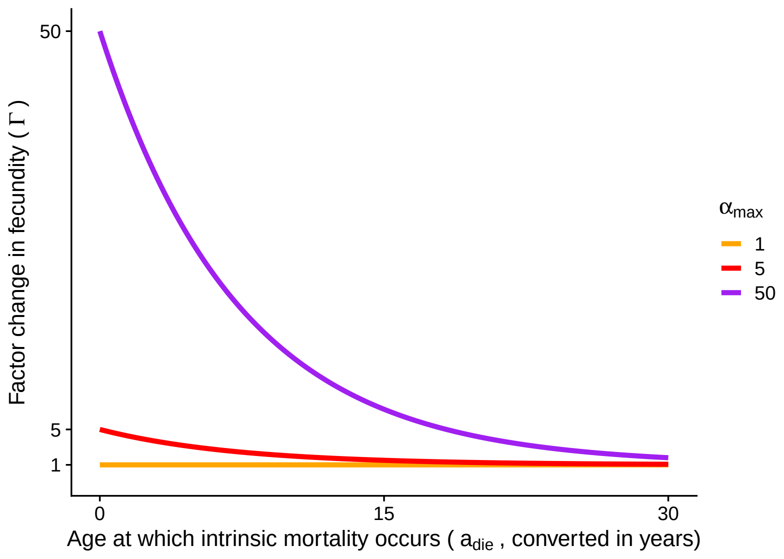

Figure S1: Factor change in fecundity depending on the age at which intrinsic mortality occurs, for different $\alpha_{\max}$. Here, $\gamma = 0.14$.

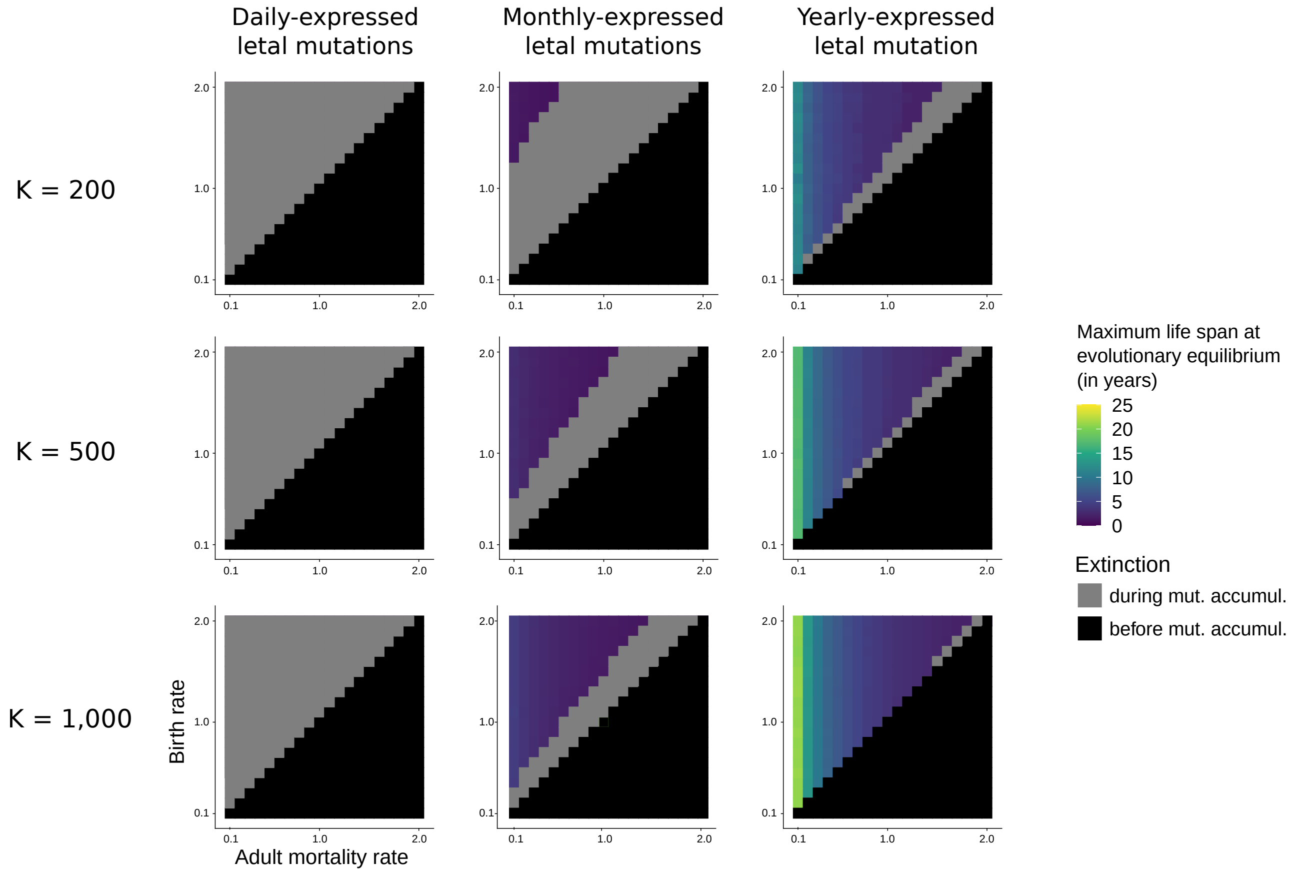

Figure S2: Maximum life span in population of carrying capacity K after 100,000 years with accumulation of non-pleiotropic lethal mutations that are either daily-, monthly-, or yearly-expressed (i.e., that can be expressed at the onset of each day, each month, or each year, respectively). Populations are characterized by birth rates and adult mortality rates spanning from 0.1 to 2. The maximum life span corresponds here to the 97.5th percentile of individual life spans.

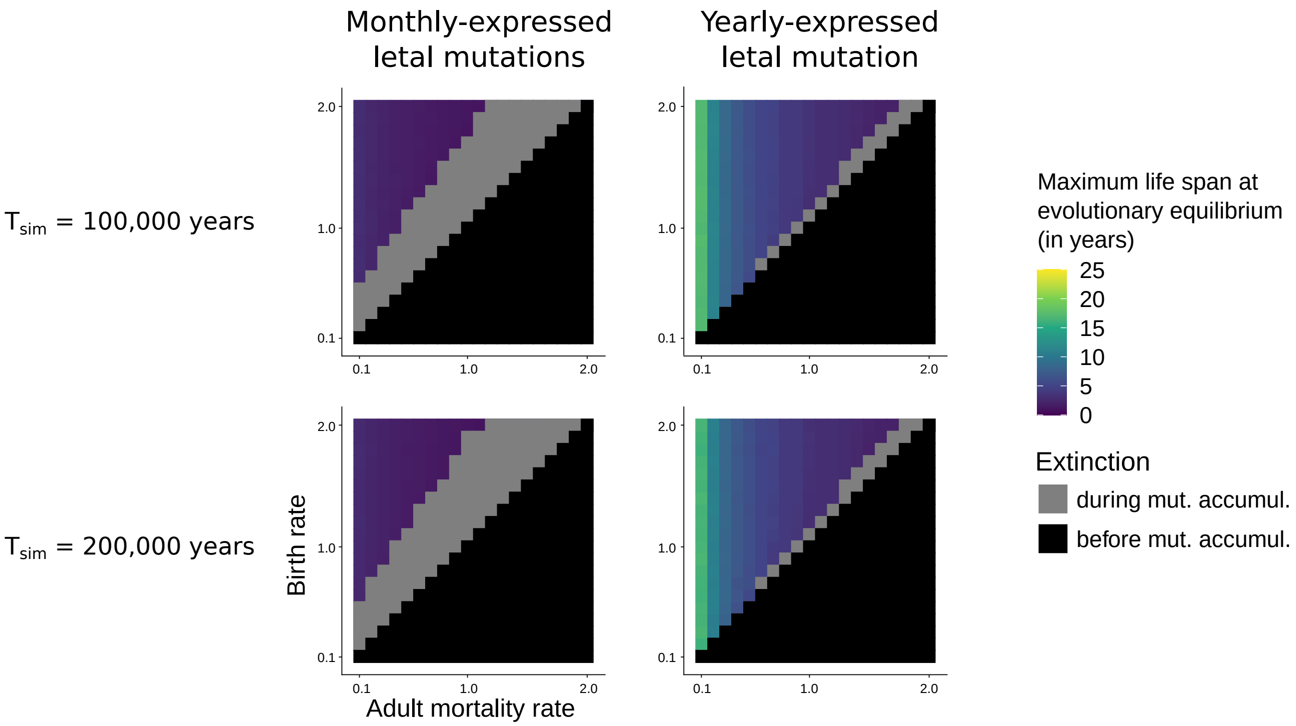

Figure S3: Maximum life span in population of carrying capacity $K = 500$ after T_{sim} years with accumulation of lethal mutations that are yearly-expressed (i.e., expressed at the onset of each year).

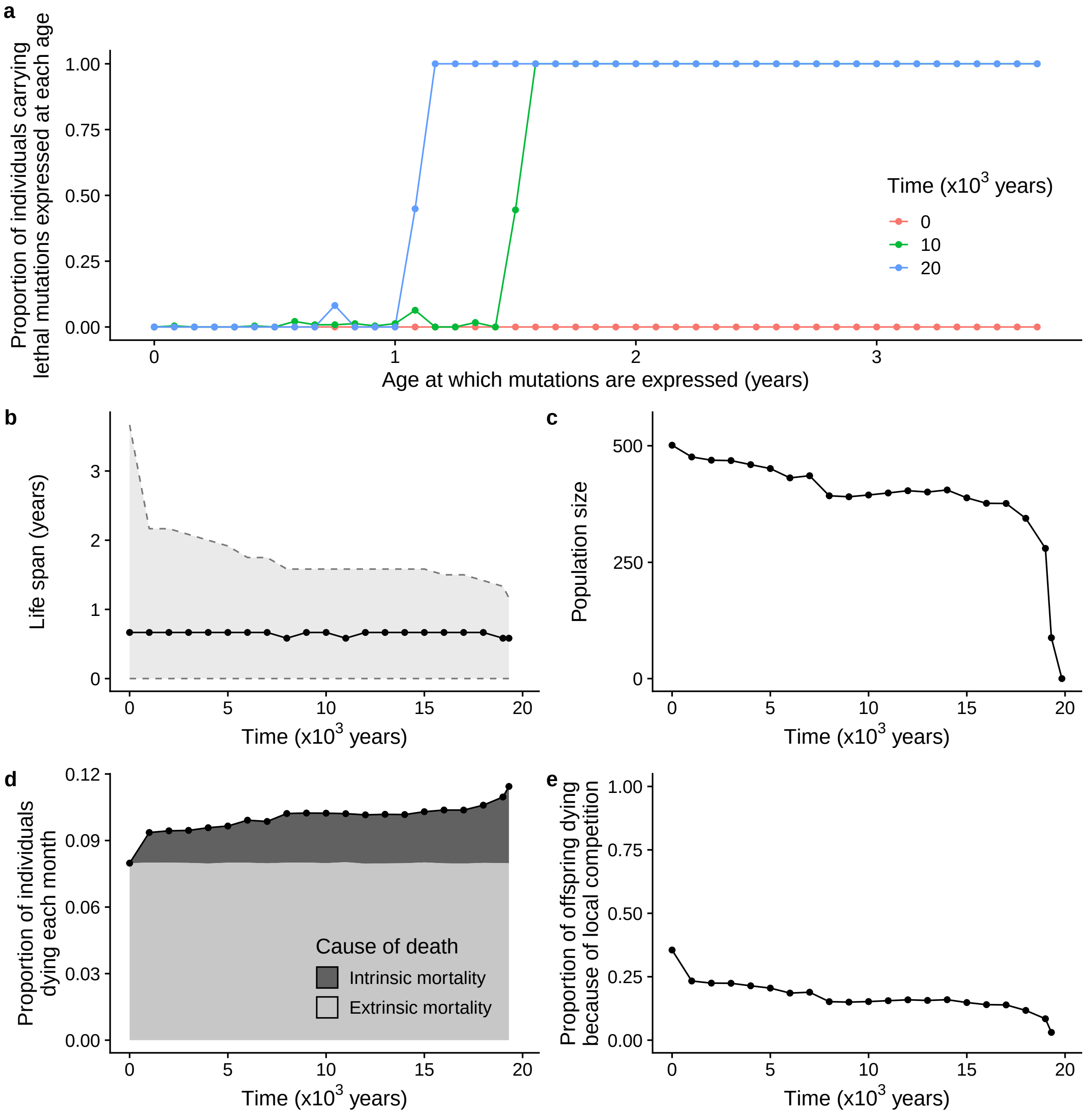

Figure S4: Simulation with a population of carrying capacity $K = 500$ and with accumulation of non-pleiotropic lethal mutations that are monthly-expressed (i.e., that can be expressed at the onset of each month). (a) Proportion of individuals that carry lethal mutations expressed at each age. (b) Evolution of the life span over time (2.5th, 50th and 97.5th percentile of individual life spans). (c) Change in population size over time. (d) Proportion of individuals dying each month, and cause of their death (extrinsic mortality, or intrinsic mortality caused by a lethal mutation). (e) Proportion of offspring individuals that are not recruited in the population, i.e., those that die because of density-dependent local competition. Here, $b_0 = 1.5$ and $\mu = 1.0$. The accumulation of monthly-expressed lethal mutations leads to senescence at an increasingly younger age, which ultimately leads population crash.

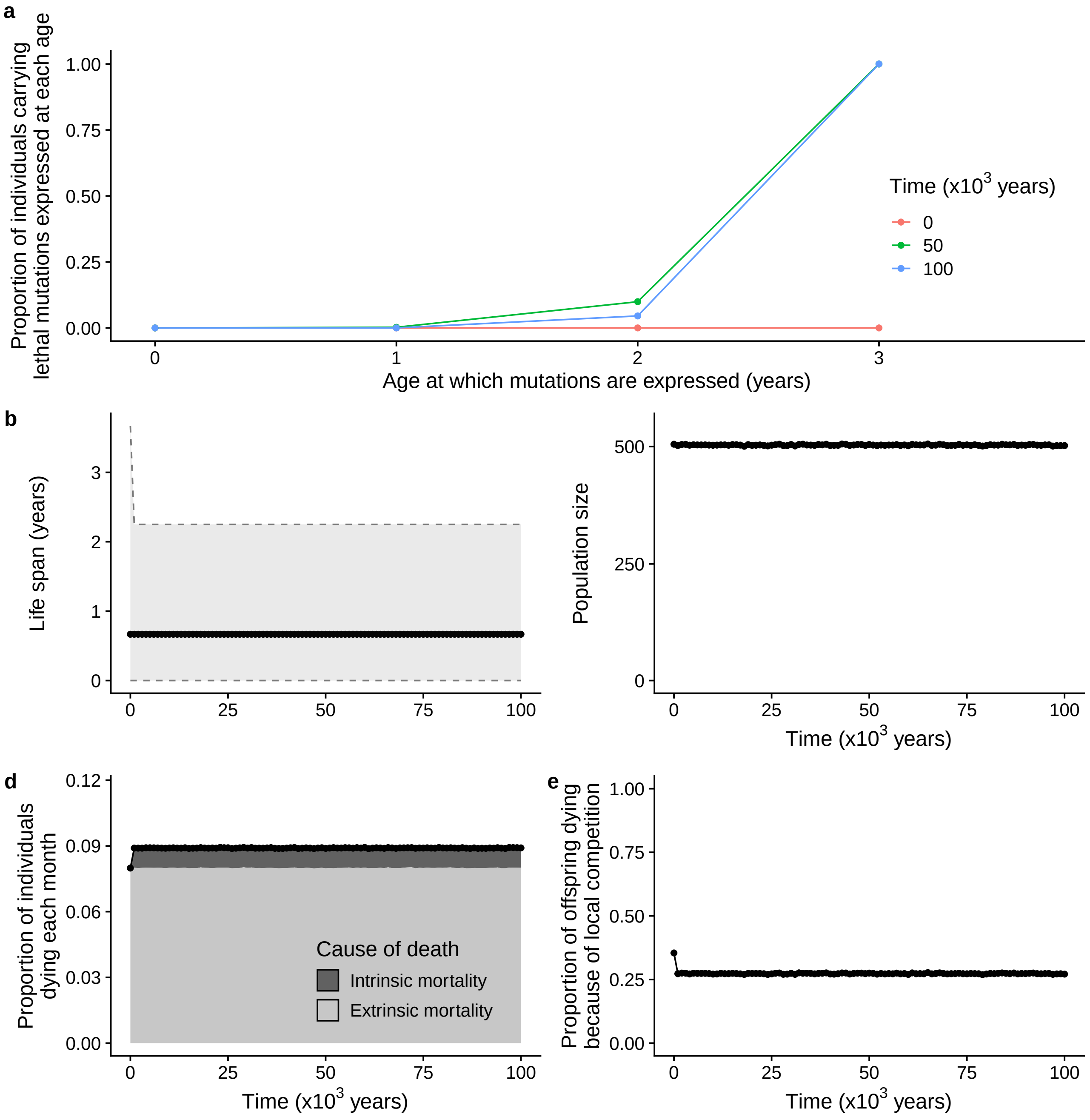

Figure S5: Simulation with a population of carrying capacity $K = 500$ and with accumulation of non-pleiotropic lethal mutations that are yearly-expressed (i.e., that can be expressed at the onset of each year). See caption of Figure S4 for details. Here, $b_0 = 1.5$ and $\mu = 1.0$. The accumulation of yearly-expressed lethal mutations leads to senescence at an increasingly younger age, but the accumulation is stopped thanks to the coarse grain of the age dependence of mutation expression, avoiding population crash.

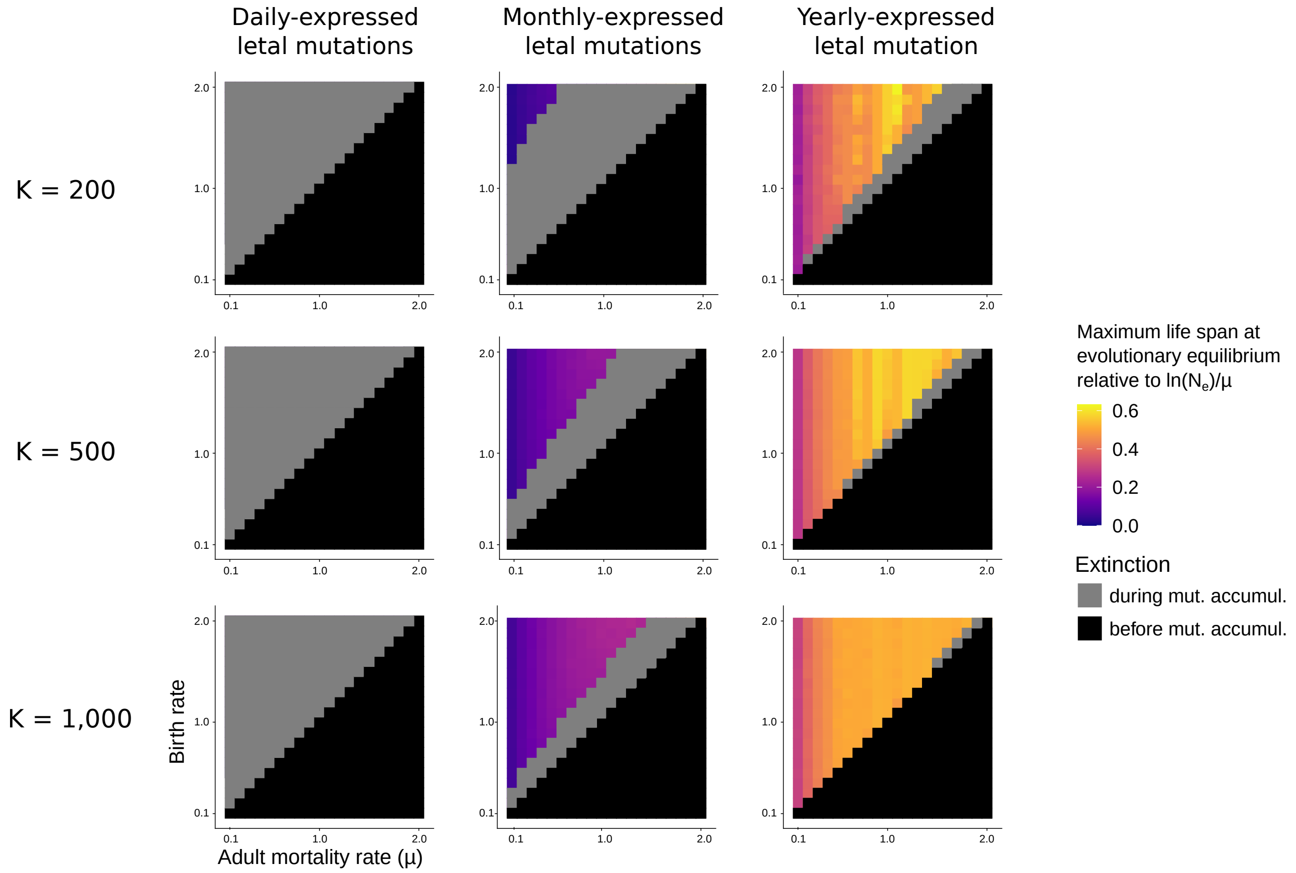

Figure S6: Maximum life span relative to $\ln(N_e)/\mu$ in population of carrying capacity $K = 500$ after T_{sim} years with accumulation of lethal mutations that are either monthly-, yearly- or biennial-expressed (i.e., that can be expressed at the onset of each month, each year, or each second year, respectively). Populations are characterized by birth rates and adult mortality rates spanning from 0.1 to 2. The maximum life span corresponds here to the 97.5th percentile of individual life spans.

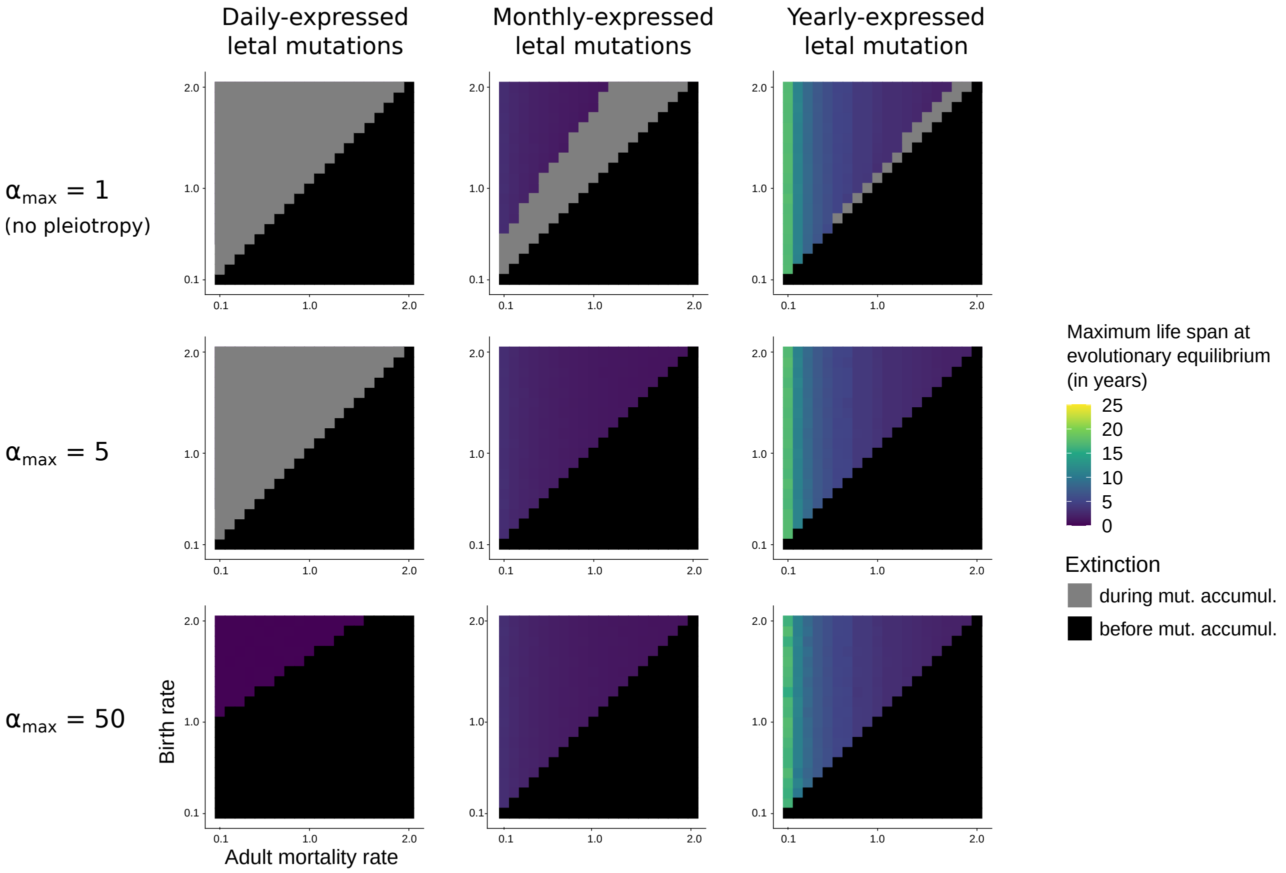

Figure S7: Maximum life span in population of carrying capacity $K = 500$ after 100,000 years with accumulation of pleiotropic lethal mutations that are either daily-, monthly-, or yearly-expressed (i.e., that can be expressed at the onset of each day, each month, or each year, respectively). Lethal mutations increase fecundity at most by a factor $\alpha_{\max} = 1$ (non-pleiotropic), $= 5$ or $= 50$. Populations are characterized by birth rates and adult mortality rates spanning from 0.1 to 2. The maximum life span corresponds here to the 97.5th percentile of individual life spans.

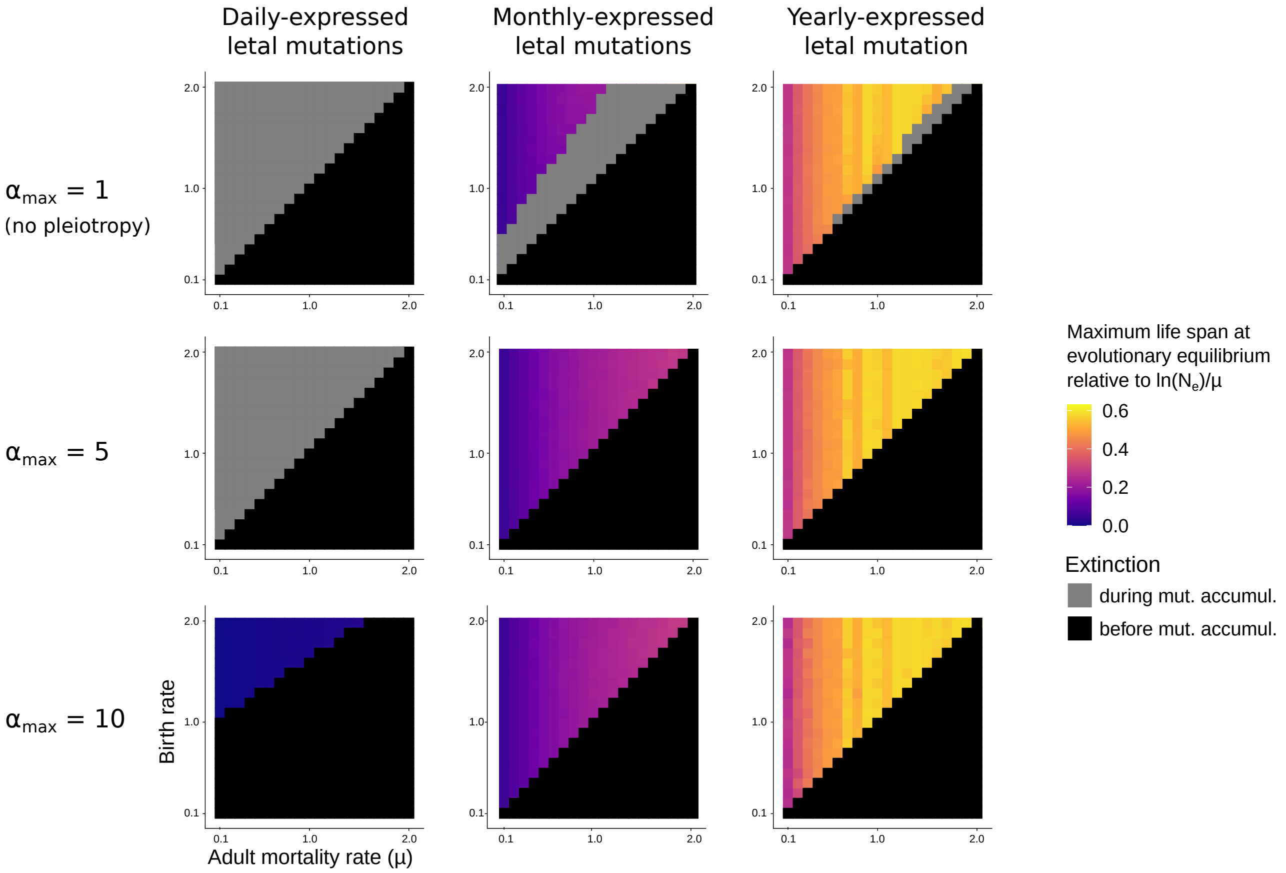

Figure S8: Maximum life span relative to $\ln(N_{\rm e})/\mu$ in population of carrying capacity $K = 500$ after 100,000 years with accumulation of pleiotropic lethal mutations that are either monthly-, yearly- or biennial-expressed (i.e., that can be expressed at the onset of each month, each year, or each second year, respectively). Lethal mutations increase fecundity at most by a factor $\alpha_{\rm max} = 1$ (non-pleiotropic),$= 5$ or $= 50$. Populations are characterized by birth rates and adult mortality rates spanning from 0.1 to 2. The maximum life span corresponds here to the 97.5th percentile of individual life spans.

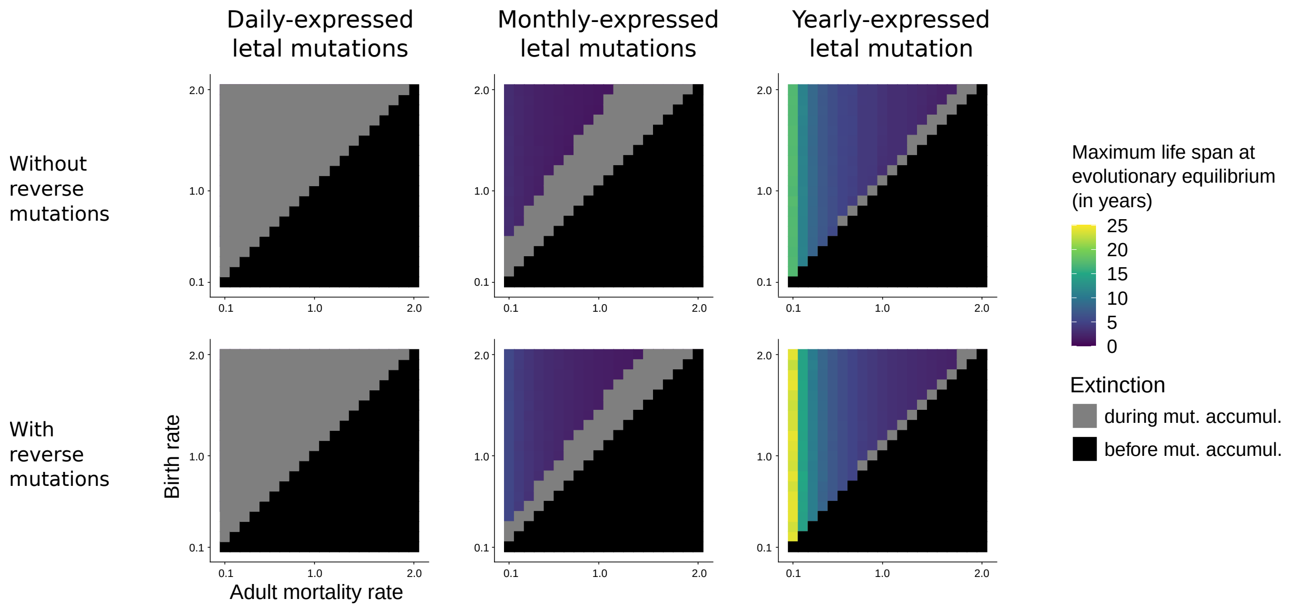

Figure S9: Maximum life span in population of carrying capacity $K = 500$ after 100,000 years with accumulation of pleiotropic lethal mutations that are either monthly-, yearly- or biennial-expressed (i.e., that can be expressed at the onset of each month, each year, or each second year, respectively). In the bottom row, we assume that reverse mutations may remove the deleterious effect of any lethal mutation with the same probability as the probability of lethal mutations to appear ($m_{\text{rev}} = m$). Populations are characterized by birth rates and adult mortality rates spanning from 0.1 to 2. The maximum life span corresponds here to the 97.5th percentile of individual life spans.

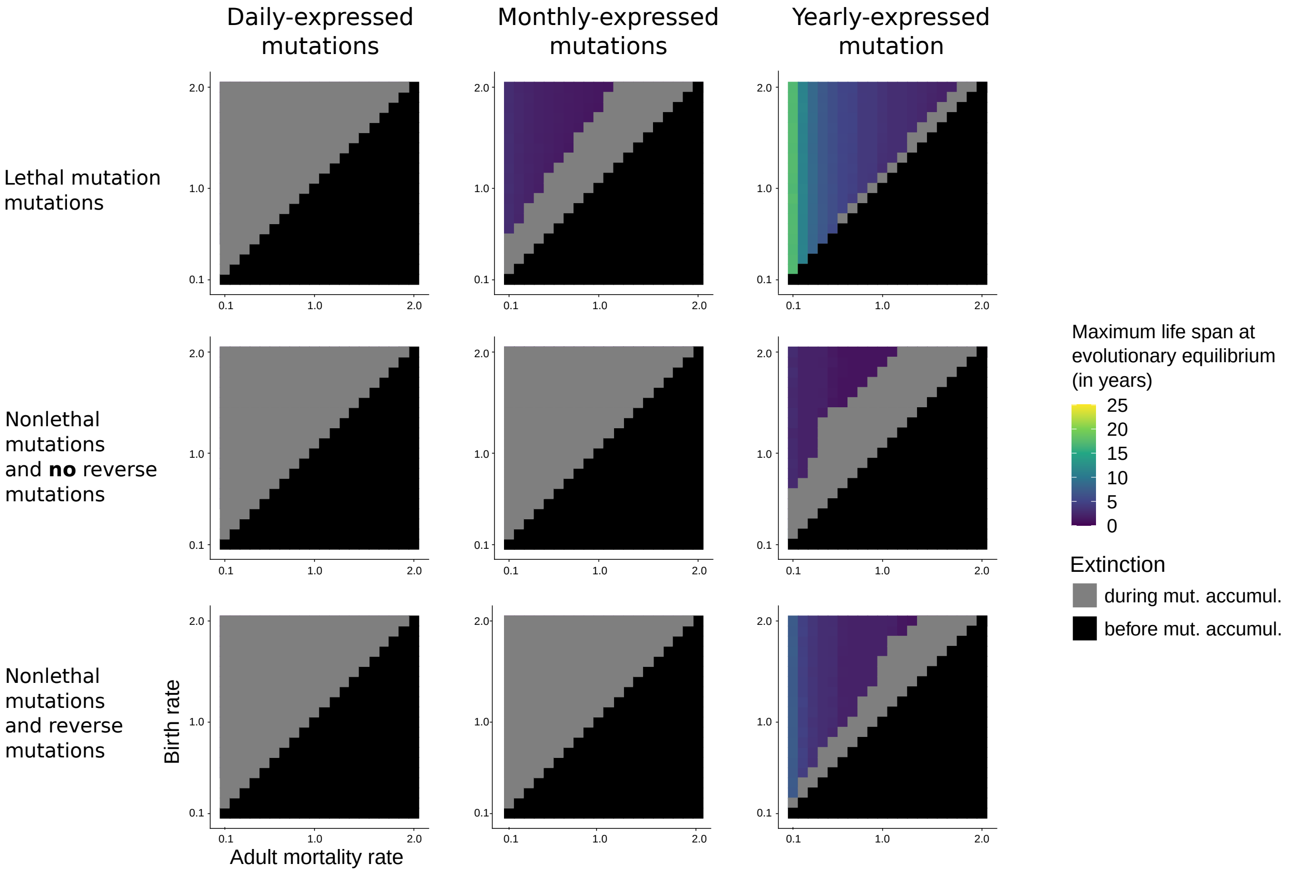

Figure S10: Maximum life span in population of carrying capacity $K = 500$ after 100,000 years with accumulation of lethal or nonlethal mutations that are either daily-, monthly- or yearly-expressed (i.e., that can be expressed at the onset of each day, each month, or each year, respectively). In the bottom row, we assume that reverse mutations may remove the deleterious effect of any nonlethal mutation with the same probability as the probability of nonlethal mutations to appear ($m_{\text{rev}} = m$). Populations are characterized by birth rates and adult mortality rates spanning from 0.1 to 2. The maximum life span corresponds here to the 97.5th percentile of individual life spans.

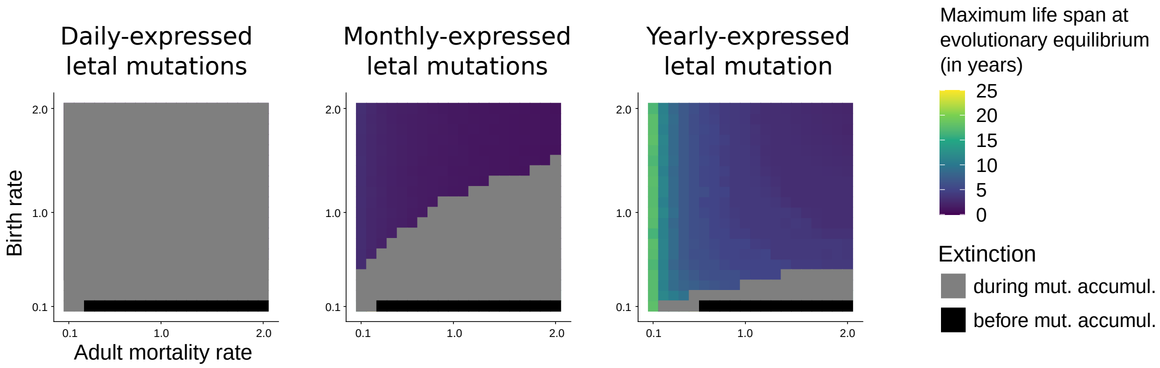

Figure S11: Maximum life span in population of carrying capacity $K = 500$ after 100,000 years with density-dependent adult extrinsic mortality. We here implement a density-dependent extrinsic mortality rate, such that extrinsic mortality is reduced by a factor equal to the ratio between the population density and the carrying capacity. Lethal mutations are either monthly-, yearly- or biennial-expressed (i.e., that can be expressed at the onset of each month, each year, or each second year, respectively). Populations are characterized by birth rates and maximum adult mortality rates spanning from 0.1 to 2. The maximum life span corresponds here to the 97.5th percentile of individual life spans.

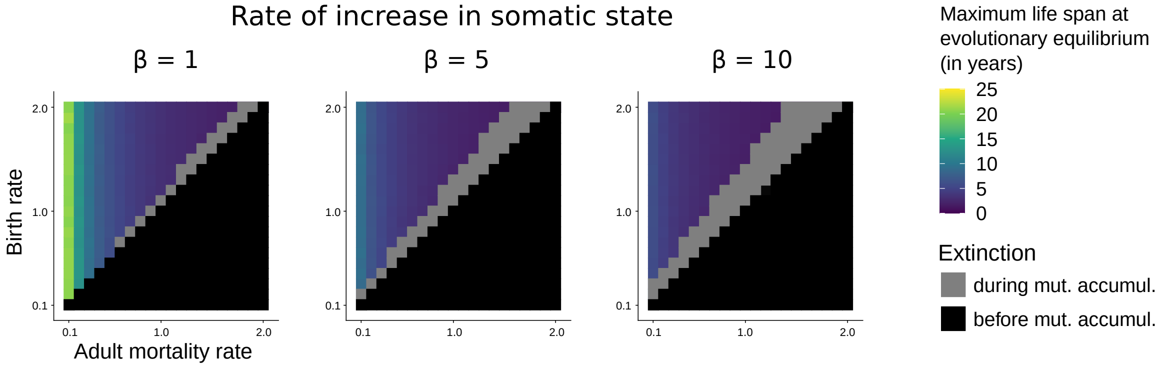

Figure S12: Maximum life span in population of carrying capacity $K = 500$ after 100,000 years with accumulation of lethal mutations that are expressed depending on the individual's somatic state. We vary the rate β at which the somatic state increase. If the somatic state changes at a high rate (high β), we get the same result is obtained as when there is a fine grain of age dependence of mutation expression. Populations are characterized by birth rates and adult mortality rates spanning from 0.1 to 2. The maximum life span corresponds here to the 97.5th percentile of individual life spans.
